# Antagonistic regulation of anthocyanin biosynthesis by HY5 and BPC1 in *Arabidopsis thaliana*

**DOI:** 10.3389/fpls.2026.1826058

**Published:** 2026-05-18

**Authors:** Dasom Choi, Hyunjoon Kim, Eojin Cho, Adji Baskoro Dwi Nugroho, Dong-Hwan Kim

**Affiliations:** Department of Plant Science and Technology, College of Biotechnology, Chung-Ang University, Anseong, Republic of Korea

**Keywords:** anthocyanin, BPC1, H3K27ac, H3K27me3, HY5, polycomb repressive complex (PRC)

## Abstract

Anthocyanins are flavonoid pigments that contribute to plant stress tolerance and environmental acclimation, yet how transcriptional activation is balanced with chromatin-based repression to fine-tune anthocyanin biosynthesis remains poorly understood. Here, we identify BASIC PENTACYSTEINE 1 (BPC1) as a key epigenetic repressor of anthocyanin biosynthesis in *Arabidopsis thaliana*. Transcriptome profiling of the *bpc1-1* mutant revealed strong upregulation of anthocyanin biosynthetic and regulatory genes, accompanied by increased anthocyanin accumulation. Analysis of a genome-wide H3K27me3 ChIP-seq dataset in Col-0 wild type showed that many anthocyanin pathway genes are substantially enriched for H3K27me3 and are directly regulated by the Polycomb Repressive Complex 2 (PRC2), including LHP1. BPC1 is required for proper H3K27me3 deposition, thereby maintaining transcriptional repression under non-inductive conditions. In contrast, BPC1 is dispensable for PRC1-mediated H2A mono-ubiquitination at these loci. We further demonstrate that the light-responsive transcription factor, ELONGATED HYPOCOTYL 5 (HY5) directly associates with multiple regulatory and biosynthetic genes in the anthocyanin pathway and promotes their expression primarily through coordination of histone acetylation, rather than by altering H3K27me3 levels. Loss of HY5 leads to reduced histone acetylation and attenuated anthocyanin accumulation without broadly affecting PRC2-mediated repression. Together, our findings support a model in which anthocyanin biosynthesis is governed by a constitutive antagonistic interplay between BPC1-mediated PRC2 repression and HY5-driven transcriptional activation under non-stressed conditions. This basal chromatin regulatory state likely serves as a molecular pre-set for rapid induction of the anthocyanin pathway in response to environmental stimuli such as UV-B radiation, providing new insight into the epigenetic coordination of secondary metabolism in plants.

## Introduction

Anthocyanins are flavonoid pigments that contribute to photoprotection, oxidative stress tolerance, and environmental acclimation in plants (Gould, 2004; Jaakola, 2013). In *Arabidopsis thaliana*, anthocyanin biosynthesis is primarily regulated at the transcriptional level through coordinated control of structural biosynthetic genes and upstream transcription factors, particularly R2R3-type MYB (Myeloblastosis) transcription factors (Petroni and Tonelli, 2011). Anthocyanin accumulation is highly responsive to environmental cues such as light quality and UV-B radiation, underscoring the importance of regulatory mechanisms that integrate external signals with endogenous transcriptional control (Xiangzhao et al., 2026).

Expression of Early Biosynthetic Genes (EBGs) and Late Biosynthetic Genes (LBGs) is positively modulated by a ternary protein complex comprising basic helix-loop-helix (bHLH) TFs, R2R3-type MYB transcription factors (TFs), and WD40 repeat proteins, referred to as MYB-bHLH-WD40 (MBW) complex (Hichri et al., 2011; Petroni and Tonelli, 2011). Several R2R3-type MYB TFs such as MYB11, MYB12, MYB111, are involved in the expression of EBGs, whereas MYB75/PAP1, and MYB90/PAP2 forms MBW complex to regulate expression of LBGs. bHLH factors including TT8 physically interact with the R3 domain of R2R3-type MYB TFs and directly bound to the promoters of anthocyanin biosynthetic genes (Yang et al., 2022). Among MBW complex, WD40 domain protein named TTG1 stabilizes the physical association between the MYB TFs and bHLH TFs (Petroni and Tonelli, 2011).

A bZIP transcription factor, ELONGATED HYPOCOTYL 5 (HY5) positively regulates anthocyanin biosynthesis by directly activating the R2R3-MYB transcription factor, *MYB75/PAP1*, one of upstream regulator of anthocyanin pathway genes (Shin et al., 2013). In addition, UV-B exposure activates UV RESISTANCE LOCUS 8 (UVR8)-dependent signaling pathways that promote HY5 accumulation and activity through modulation of COP1 function, thereby enhancing transcriptional induction of downstream anthocyanin pathway genes (Rizzini et al., 2011; Favory et al., 2009; Jenkins, 2017).

Epigenetic regulation represents a fundamental layer of transcriptional control in plants, with Polycomb Repressive Complex 2 (PRC2) mediating stable gene silencing through deposition of a repressive histone mark, H3K27me3 on target chromatin (Mozgova et al., 2015). Epigenetic repression in plants is mediated in large part by two functionally coordinated Polycomb group protein complexes, Polycomb Repressive Complex 2 (PRC2) and Polycomb Repressive Complex 1 (PRC1). In *Arabidopsis*, the catalytic core of PRC2 is composed of the histone methyltransferases CURLY LEAF (CLF) and SWINGER (SWN), which are homologs of the *Drosophila* E(z) protein, together with the essential subunits FERTILIZATION INDEPENDENT ENDOSPERM (FIE), MULTICOPY SUPPRESSOR OF IRA 1 (MSI1), and either EMBRYONIC FLOWER 2 (EMF2), VERNALIZATION 2 (VRN2), or FERTILIZATION INDEPENDENT SEED 2 (FIS2), which define distinct PRC2 sub-complexes (Mozgova and Hennig, 2015; Kim and Sung, 2014). PRC2-mediated H3K27me3 is broadly distributed across the *Arabidopsis* genome, covering thousands of developmental and stimulus-responsive genes, and its establishment is a prerequisite for stable transcriptional repression at many loci (Bouyer et al., 2011; Mozgova et al., 2015). A PRC2-associated protein, LIKE HETEROCHROMATIN PROTEIN 1 (LHP1), binds H3K27me3 marks both *in vitro* and *in vivo* and is required for a functional Polycomb group system in plants (Turck et al., 2007). MSI1, a core subunit of all PRC2 complexes in *Arabidopsis*, directly interacts with LHP1 and thereby physically connects LHP1 to PRC2. This LHP1-MSI1 interaction forms a positive feedback loop to recruit PRC2 to chromatin already carrying H3K27me3, providing a mechanism for the faithful maintenance and inheritance of H3K27me3 at target loci (Derkacheva et al., 2013). Furthermore, LHP1 is responsible for the spreading of H3K27me3 towards the 3′ end of the gene body at PRC2 target loci, a function that depends on LHP1 and the PRC2 subunit, CLF (Veluchamy et al., 2016). Collectively, these findings indicate that LHP1 functions with PRC2 to establish and maintain H3K27me3, and that LHP1–PRC2 interactions facilitate the recruitment of PRC2 to target genes, making LHP1 and PRC2 closely integrated components of the Polycomb repression machinery in *Arabidopsis* (Derkacheva et al., 2013; Wang et al., 2017).

PRC1 acts coordinately with PRC2 to reinforce Polycomb-mediated gene silencing. In *Arabidopsis*, PRC1 comprises five RING-finger proteins, ARABIDOPSIS THALIANA RING 1A (AtRING1A) and ARABIDOPSIS THALIANA RING 1B (AtRING1B) (homologs of mammalian RING1) and ARABIDOPSIS B LYMPHOMA MO-MLV INSERTION REGION 1 HOMOLOG A (AtBMI1A), ARABIDOPSIS B LYMPHOMA MO-MLV INSERTION REGION 1 HOMOLOG B (AtBMI1B), and ARABIDOPSIS B LYMPHOMA MO-MLV INSERTION REGION 1 HOMOLOG C (AtBMI1C) (homologs of mammalian BMI1/PCGF) which are the catalytic subunits responsible for H2A monoubiquitination (H2Aub1), a hallmark of PRC1 catalytic activity (Bratzel et al., 2010; Chen et al., 2010; Yang et al., 2013). PRC1-mediated H2Aub1 leads to chromatin compaction and transcriptional repression (Yang et al., 2013; Bratzel et al., 2010). The relationship between PRC1 and PRC2 is not strictly hierarchical: PRC1-mediated H2Aub1 can act upstream of PRC2 to initiate the repression of actively transcribed genes, after which PRC2-mediated H3K27me3 is established to maintain stable gene silencing (Yang et al., 2013). Conversely, genome-wide profiling analyses have revealed that PRC1 components, including AtRING1A/B and AtBMI1A/B, also contribute to the maintenance of H3K27me3 across the genome, suggesting context-dependent interdependence between the two complexes (Wang et al., 2017; Kralemann et al., 2020).

Despite significant progress in understanding the catalytic activities of PRC1 and PRC2, a central question remains: how are these complexes recruited to specific chromatin loci with sufficient selectivity to maintain gene-specific repression programs. In plants, sequence-specific DNA-binding transcription factors have emerged as key PRC2-tethering factors. Among these, the BASIC PENTACYSTEINE (BPC) family of plant-specific GAGA-motif binding proteins has been identified as one of the most significant classes of PRC2 recruiters, with all six characterized BPC members (BPC1-BPC6) implicated in PRC2 tethering in *Arabidopsis* (Godwin and Farrona, 2022). The molecular mechanisms linking BPC proteins to PRC2 have been dissected across several developmental contexts in plants (Meister et al., 2004; Hecker et al., 2015). Several BPC proteins physically interact with SWN, a core catalytic subunit of PRC2, and directly recruit PRC2 to their genomic binding sites, where it catalyzes H3K27me3 deposition to epigenetically repress target gene expression (Xiao et al., 2017). In addition, BPCs bind to GAGA-type sequences in the *ABI4* promoter and repress *ABI4* transcription in roots by recruiting PRC2 to catalyze H3K27me3 at this locus (Mu et al., 2017). Another study reported that the Class II member BPC6 recruits LHP1 to GAGA-motif-containing PREs in the promoters of homeotic genes *in vitro*, and this BPC6-LHP1 complex is proposed to associate with the PRC2 component VRN2 *in vivo* to establish H3K27me3 (Hecker et al., 2015). The functional link between BPC proteins and PRC2 further extends to reproductive development, where Class I BPC1 and BPC2 interact with the FERTILIZATION INDEPENDENT SEED PRC2 (FIS-PRC2) sub-complex to repress *FUSCA3* (*FUS3*) expression in the endosperm, thereby coordinating early endosperm and embryo growth after fertilization (Wu et al., 2020). Consistent with these findings, both Class I and Class II BPCs act redundantly to confine the expression of the ovule identity gene *SEEDSTICK* (*STK*) in floral tissues, most likely by recruiting LHP1 and mediating the establishment and maintenance of H3K27me3 repressive marks at the STK locus (Petrella et al., 2020). Collectively, these cumulative studies establish the BPC-PRC2 axis as a conserved and broadly deployed mechanism for sequence-specific epigenetic silencing of developmental gene programs in *Arabidopsis* (Theune et al., 2019).

Whether this mechanism extends beyond developmental genes to regulate environmentally responsive secondary metabolic pathways such as anthocyanin biosynthesis has, however, remained unexplored. In this study, transcriptome profiling of the *bpc1–1* mutant revealed significant enrichment of anthocyanin-related category in gene ontology (GO) analysis, indicating a previously unrecognized role for BPC1 in the regulation of anthocyanin pathway gene expression. Integrating this finding with the established function of HY5 as a light- and UV-B-responsive transcriptional activator, our results support a model in which anthocyanin biosynthesis is governed by an antagonistic interplay between inducible transcriptional activation and BPC1-mediated chromatin-based repression.

## Results

### Transcriptome analysis indicated that BPC1 might be involved in the control of secondary metabolites like anthocyanin biosynthesis

**Figure 1:**
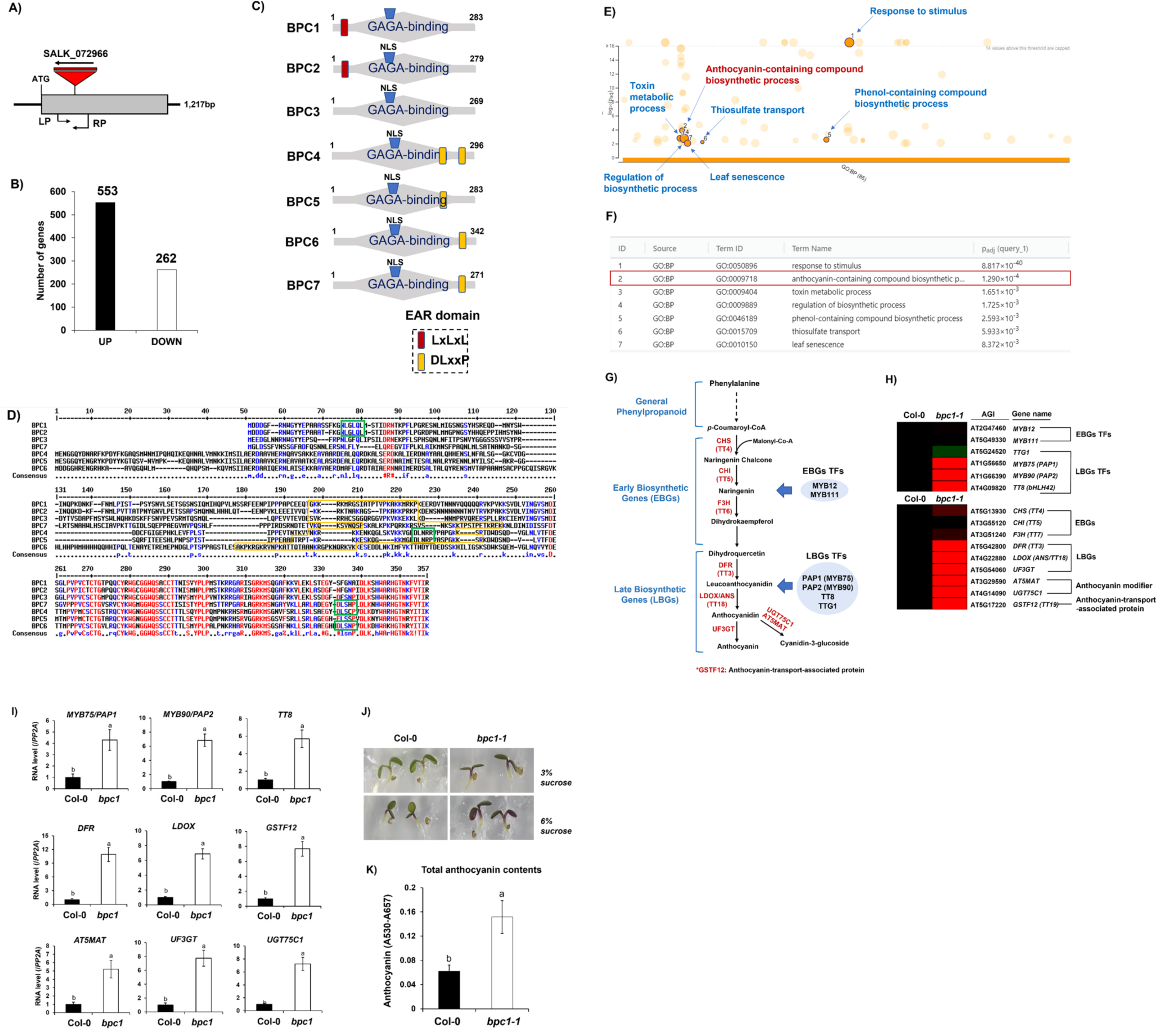
Anthocyanin content was highly increased in the bpc1–1 mutant compared to that of Col-0. **(A)** A diagram showing the location of T-DNA insertion in the coding region (indicated with gray box) of BPC1 gene. ATG: start codon, LP: left primer for genotyping, RP: right primer for genotyping. The *bpc1–1* mutant (SALK_072966) was obtained from the *Arabidopsis* Information Resource (TAIR). **(B)** a bar graph showing up- and down-regulated genes in the *bpc1–1* mutant in comparison to those of Col-0. **(C)** Schematic presentation of seven BASIC PENTACYSTEINE (BPC) family proteins in *Arabidopsis*. EAR domains (-LxLxLx-/-DLxxP-) located in the BPC family proteins were indicated with red or orange color box. **(D)** Multiple sequence alignment of seven BPC family proteins in *Arabidopsis*. Red, blue, and black color letters indicate high consensus, low consensus, and neutral amino acids among BPC family proteins in *Arabidopsis*. EAR domains (-LxLxLx-/-DLxxP-) were indicated with green box. Overall, C-terminal region was highly conserved in BPC family proteins. **(E)** Result of gene ontology (GO) analysis using 553 upregulated genes in *bpc1–1* mutant. Top ranked GO terms were indicated with green color circles. GO terms related to biosynthesis related to secondary defensive compounds like ‘anthocyanin-containing compound biosynthesis’, ‘Phenol-containing compound biosynthetic process’, ‘Toxin metabolic process’, and ‘Regulation of biosynthetic process’ were substantially detected in the top ranked terms. GO analysis was performed using the g:Profiler software (https://biit.cs.ut.ee/gprofiler/gost). **(F)** List of top 7 ranked GO terms using 553 upregulated genes in *bpc1–1* mutant in comparison to those of Col-0 wild type. A GO term related to the anthocyanin-containing compound biosynthesis was indicated with red box. **(G)** Schematic diagram showing biosynthetic process of anthocyanin and its related proteins. Red letters indicate the biosynthetic enzymes responsible for catalytic conversion. Biosynthetic genes for anthocyanin production can be grouped into two groups: early biosynthetic genes (EBGs) and late biosynthetic genes (LBGs). Blue circles indicate transcription factors responsible for the regulation of EBGs (*MYB12* and *MYB111*) and LBGs (*MYB75/PAP1, MYB90/PAP2, TT8*, and *TTG1*). **(H)** A heatmap showing normalized RNA-seq transcript reads of 15 anthocyanin pathway genes between Col-0 and *bpc1–1* mutant. Nine genes out of total 15 anthocyanin pathway genes exhibited substantially upregulated expression in *bpc1–1* mutant compared to those of Col-0. Each block of value represents the mean of three replicates. The mean value of Col-0 was normalized to 1. Green to red color represents low to high transcript levels. **(I)** Result of quantitative RT-PCR (qRT-PCR) analysis on nine anthocyanin pathway genes between Col-0 and *bpc1–1* mutant. All tested nine anthocyanin pathway genes exhibited significantly upregulated expression in *bpc1–1* mutant compared to those of Col-0. One-way ANOVA was applied to calculate the statistical significance, and significant difference was indicated in the figures by different letters (p < 0.05). **(J)** Representative image showing amounts of anthocyanin between Col-0 and *bpc1–1* mutant grown for one week in the Murashige and Skoog (MS) agar media containing 3% or 6% sucrose. **(K)** Quantification of anthocyanin between Col-0 and *bpc1–1* mutant grown for one week in the Murashige and Skoog (MS) agar media containing 3% sucrose. One-way ANOVA was applied to calculate the statistical significance, and significant difference was indicated in the figures by different letters (p < 0.05).

To capture the functional role of BPC1, we isolated a functional knock-out mutant of *BPC1* (named as *bpc1-1*) (**Figure 1A**). RNA-seq analysis was conducted between Col-0 and *bpc1–1* mutant at the two weeks old seedling stage. As a result, total 815 differentially expressed genes (DEGs) were identified between Col-0 and *bpc1-1*. Total 553 and 262 genes were up- and down-regulated in *bpc1–1* mutant, respectively (**Figure 1B**; **Supplementary Figure S1**). Number of upregulated genes in *bpc1–1* was more than 2 times of number of downregulated genes (**Figure 1B**). Regarding this observation, BPC1 possesses an EAR domain at the N-terminal region, thus functioning as a transcriptional repressor (**Figures 1C, D**). We reasoned that list of upregulated genes in *bpc1–1* mutant might contain direct target genes. Therefore, we performed gene ontology (GO) analysis using 553 upregulated genes in *bpc1–1* mutant. Interestingly, biosynthesis related to secondary defensive compounds like ‘anthocyanin-containing compound biosynthesis’, ‘Phenol-containing compound biosynthetic process’, ‘Toxin metabolic process’, and ‘Regulation of biosynthetic process’ were significantly included in the top ranked terms (**Figures 1E, F**).

### Anthocyanin biosynthetic genes are upregulated in *bpc1–1* mutant compared to *Col-0*

Anthocyanin biosynthesis is orchestrated by a coordinated regulatory network involving transcription factors and downstream biosynthetic genes. This network includes early biosynthetic gene (EBG) regulators such as *MYB12* and *MYB111*, late biosynthetic gene (LBG) regulators including *MYB75/PAP1, MYB90/PAP2, TT8/bHLH42*, and *TTG1* (**Figure 1G**). The downstream genes comprise EBGs (*CHS/TT4*, *CHI/TT5*, and *F3H/TT6*), LBGs (*DFR/TT3, LDOX/ANS/TT18*, and *UF3GT*), anthocyanin-modifying enzymes (*UGT75C1* and *AT5MAT*), and a transport-associated protein (*GSTF12*). Because GO analysis of genes upregulated in the *bpc1–1* mutant revealed significant enrichment of defense-related secondary metabolites like anthocyanin biosynthesis, we examined the transcript levels of anthocyanin biosynthetic pathway genes in *Col-0* and the *bpc1–1* mutant. Consistent with the GO analysis, anthocyanin pathway genes were significantly upregulated in the *bpc1–1* mutant compared with *Col-0* (**Figure 1H**). Notably, 9 out of 15 anthocyanin-related genes, *MYB75/PAP1, MYB90/PAP2, TT8, DFR, LDOX, UF3GT, AT5MAT, UGT75C1*, and *GSTF12* showed markedly higher expression levels in the *bpc1–1* mutant compared to those of Col-0 wild type. To validate the RNA-seq results, we performed RT–qPCR analysis in *Col-0* and *bpc1-1*. In agreement with the transcriptome data, the same nine anthocyanin pathway genes were significantly upregulated in the *bpc1–1* mutant relative to Col-0 (**Figure 1I**). These results indicate that BPC1 negatively regulates the expression of genes involved in anthocyanin biosynthesis in *Arabidopsis*. Finally, anthocyanin content was quantified in *Col-0* and the *bpc1–1* mutant. Consistent with the RNA-seq and RT–qPCR data, anthocyanin accumulation was significantly higher in the *bpc1–1* mutant than in *Col-0* (**Figures 1J, K**). Taken together, these results demonstrate that BPC1 functions as a repressor of anthocyanin biosynthesis in *Arabidopsis*.

### Anthocyanin pathway genes can be classified into H3K27me3-enriched and H3K27me3-depleted groups

**Figure 2:**
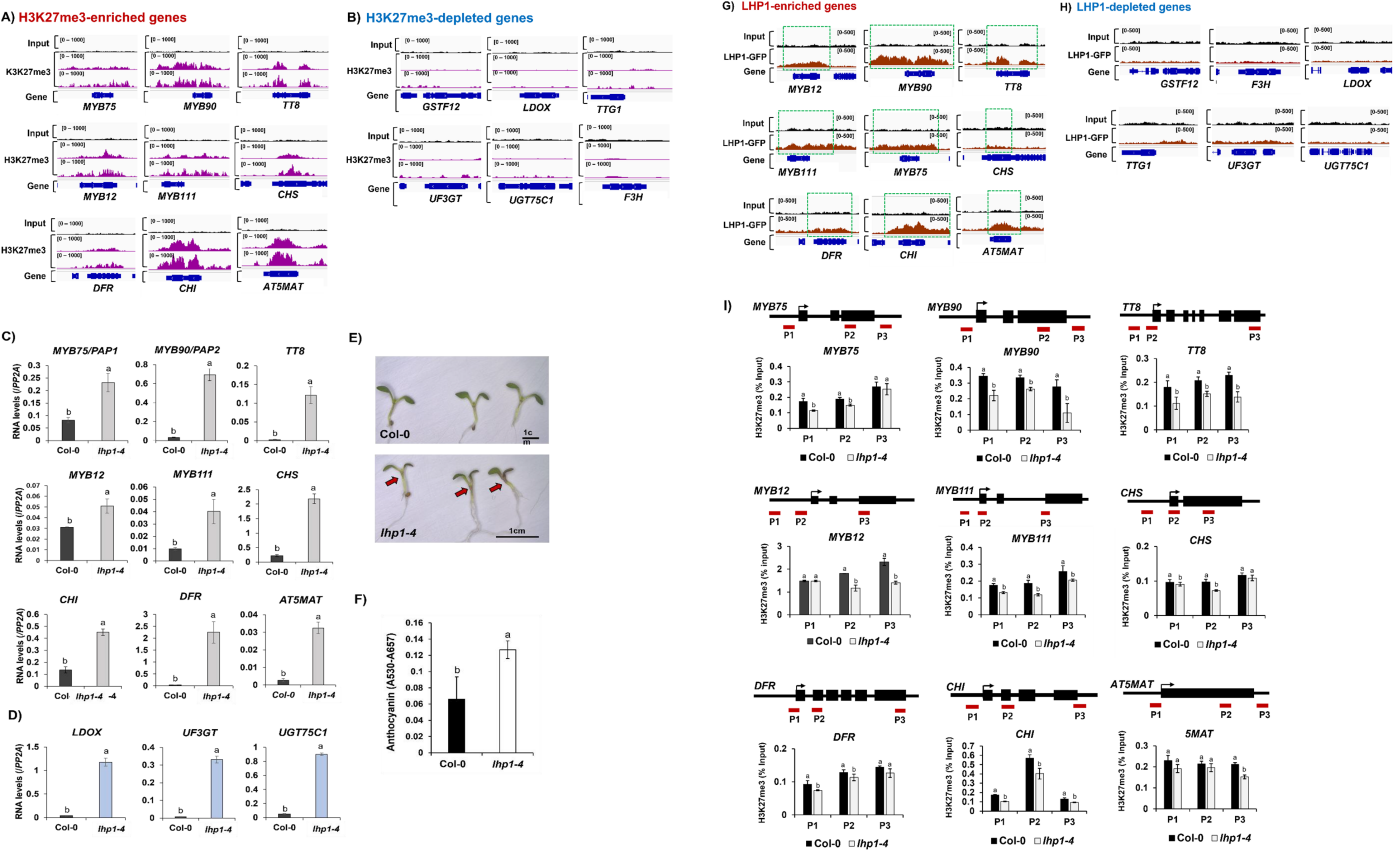
Anthocyanin pathway genes are negatively regulated by polycomb repressive complex 2 (PRC2) in Arabidopsis. **(A)** Genomic browser view illustrations showing aligned ChIP-seq reads of nine H3K27me3-enriched anthocyanin pathway genes in *Arabidopsis*. Aligned ChIP-seq reads of H3K27me3 were presented with purple color. Aligned ChIP-seq reads of Input DNA was presented with black color. Compared to level of input DNA, nine anthocyanin pathway genes were significantly enriched with H3K27me3. **(B)** Genomic browser view illustrations showing six H3K27me3-depleted anthocyanin pathway genes in *Arabidopsis*. Aligned ChIP-seq reads of H3K27me3 were presented with purple color. Aligned ChIP-seq reads of Input DNA was presented with black color. Compared to level of input DNA, five anthocyanin pathway genes were merely enriched with H3K27me3. **(A, B)** Read coverage normalized by total number of mapped reads are indicated at the top left or right corner of each track in bracket. Information of public ChIP-seq dataset used in this analysis was shown in the **Supplementary Table S3**. **(C)** Result of qRT-PCR on nine H3K27me3-enriched anthocyanin pathway genes between Col-0 and *lhp1–4* mutant. Most of tested anthocyanin pathway genes exhibited upregulated expression in *lhp1–4* mutant compared to those of Col-0. **(D)** Result of qRT-PCR analysis between Col-0 and *lhp1–4* mutant on three genes (*LDOX, UF3GT*, and *UGT75C1*) among the H3K27me3-depleted anthocyanin pathway genes. All of three H3K27me3-depleted anthocyanin pathway genes were also highly upregulated in *lhp1–4* mutant compared to those of Col-0. **(C, D)** One-way ANOVA was applied to calculate the statistical significance, and significant difference was indicated in the figures by different letters (p < 0.05). **(E)** A representative image showing amounts of anthocyanin between Col-0 and *lhp1–4* mutant grown for one week in the Murashige and Skoog (MS) agar media containing 3% sucrose. Scale bar = 1cm. **(F)** Quantification of anthocyanin between Col-0 and *lhp1–4* mutant grown for one week in the Murashige and Skoog (MS) agar media containing 3% sucrose. One-way ANOVA was applied to calculate the statistical significance, and significant difference was indicated in the figures by different letters (p < 0.05). **(G)** Genomic browser view illustrations showing aligned ChIP-seq reads of LHP1-GFP on nine H3K27me3-enriched anthocyanin pathway genes between input DNA (black color) and α-GFP-immunoprecipitated DNA (red color). Significant enrichment of LHP1-GFP was indicated with dotted green box. All nine H3K27me3-enriched anthocyanin pathway genes were densely enriched with LHP1-GFP. **(H)** Genomic browser view illustrations showing aligned ChIP-seq reads of LHP1-GFP on six H3K27me3-depleted anthocyanin pathway genes between input DNA (black color) and α-GFP-immunoprecipitated DNA (red color). No significant enrichment of LHP1-GFP was observed in the six H3K27me3-depleted anthocyanin pathway genes. Read coverage normalized by total number of mapped reads are indicated at the top left or right corner of each track in bracket. Information of public ChIP-seq dataset used in this analysis was shown in the **Supplementary Table S3**. **(I)** Result of ChIP-qPCR analysis using α-H3K27me3 between Col-0 and *lhp1–4* mutant on nine H3K27me3-enriched anthocyanin pathway genes. Each result represents the mean ± standard deviation (SD) of three independent biological replicates (n=3). Different letters represent significant differences (*P* < 0.05) determined by one-way analysis of variance (ANOVA) with Tukey’s *post-hoc* test. .

It has been previously reported that BPC1 suppresses the ovule identity gene, *SEEDSTICK* (*STK*) by coordinating the deposition of a repressive histone mark, H3K27me3 on *STK* chromatin, which is catalyzed by the Polycomb Repressive Complex 2 (PRC2) (Pinyopich et al., 2003; Kooiker et al., 2005). Because BPC1 may exert a repressive function in concert with PRC2, we analyzed publicly available *Arabidopsis* H3K27me3 ChIP-seq datasets (Wang et al., 2016; Cui et al., 2016). Among the total of 15 anthocyanin pathway genes, nine genes (*MYB75/PAP1, MYB90/PAP2, TT8, MYB12, MYB111, CHS, CHI, DFR*, and *AT5MAT*) were substantially enriched for the H3K27me3 histone mark (**Figure 2A**), whereas six genes (*F3H, LDOX, TTG1, UF3GT, GSTF12*, and *UGT75C1*) showed little to no enrichment of H3K27me3 (**Figure 2B**). These results suggest that anthocyanin pathway genes can be divided into two distinct groups in *Arabidopsis*: H3K27me3-enriched and H3K27me3-depleted genes. Notably, a substantial proportion of anthocyanin pathway genes (9 out of 15, 60%) appear to be epigenetically repressed by the PRC2 complex.

### PRC2 complex containing LHP1 directly regulates H3K27me3-enriched anthocyanin pathway genes

To assess whether H3K27me3 negatively regulates these H3K27me3-enriched genes, we performed qRT-PCR analysis between Col-0 and the *lhp1–4* mutant, a knockout mutant of LIKE HETEROCHROMATIN PROTEIN 1 (LHP1), a PRC2-associated component gene in *Arabidopsis*. All nine H3K27me3-enriched genes tested (*MYB75/PAP1, MYB90/PAP2, TT8, MYB12, MYB111, CHS, CHI, DFR*, and *AT5MAT*) were markedly de-repressed in the lhp1–4 mutant compared with Col-0 (**Figure 2C**). Unexpectedly, tested three H3K27me3-depleted genes (LDOX, UF3GT, and UGT75C1) were also significantly upregulated in the lhp1–4 mutant relative to Col-0 (**Figure 2D**). Given that these downstream biosynthetic genes are transcriptionally regulated by upstream transcription factors such as MYB75/PAP1 and MYB90/PAP2, this upregulation is likely an indirect consequence of enhanced expression of upstream regulatory genes. Consistent with the qRT-PCR result, anthocyanin content was significantly higher in the *lhp1–4* mutant than in Col-0 (**Figures 2E, F**).

To further validate PRC2-mediated regulation, we analyzed publicly available ChIP-seq data generated using an LHP1-GFP transgenic line. Relative to input DNA, LHP1-GFP showed strong enrichment at all nine H3K27me3-enriched anthocyanin pathway genes (**Figure 2G**), whereas little enrichment was observed at the six H3K27me3-depleted genes (**Figure 2H**). Lastly, we performed ChIP-qPCR analysis using an anti-H3K27me3 antibody in Col-0 and lhp1–4 mutant. All nine H3K27me3-enriched anthocyanin pathway genes exhibited significantly reduced H3K27me3 levels in the *lhp1–4* mutant compared with Col-0 (**Figure 2I**). Taken together, these results demonstrate that anthocyanin pathway genes in *Arabidopsis* can be classified into nine H3K27me3-enriched genes, which are directly regulated by the LHP1-containing PRC2 complex, and six H3K27me3-depleted genes, which are likely regulated indirectly through PRC2-controlled transcription factors.

### BPC1 directly suppresses regulatory and biosynthetic genes through coordinating deposition of H3K27me3

**Figure 3:**
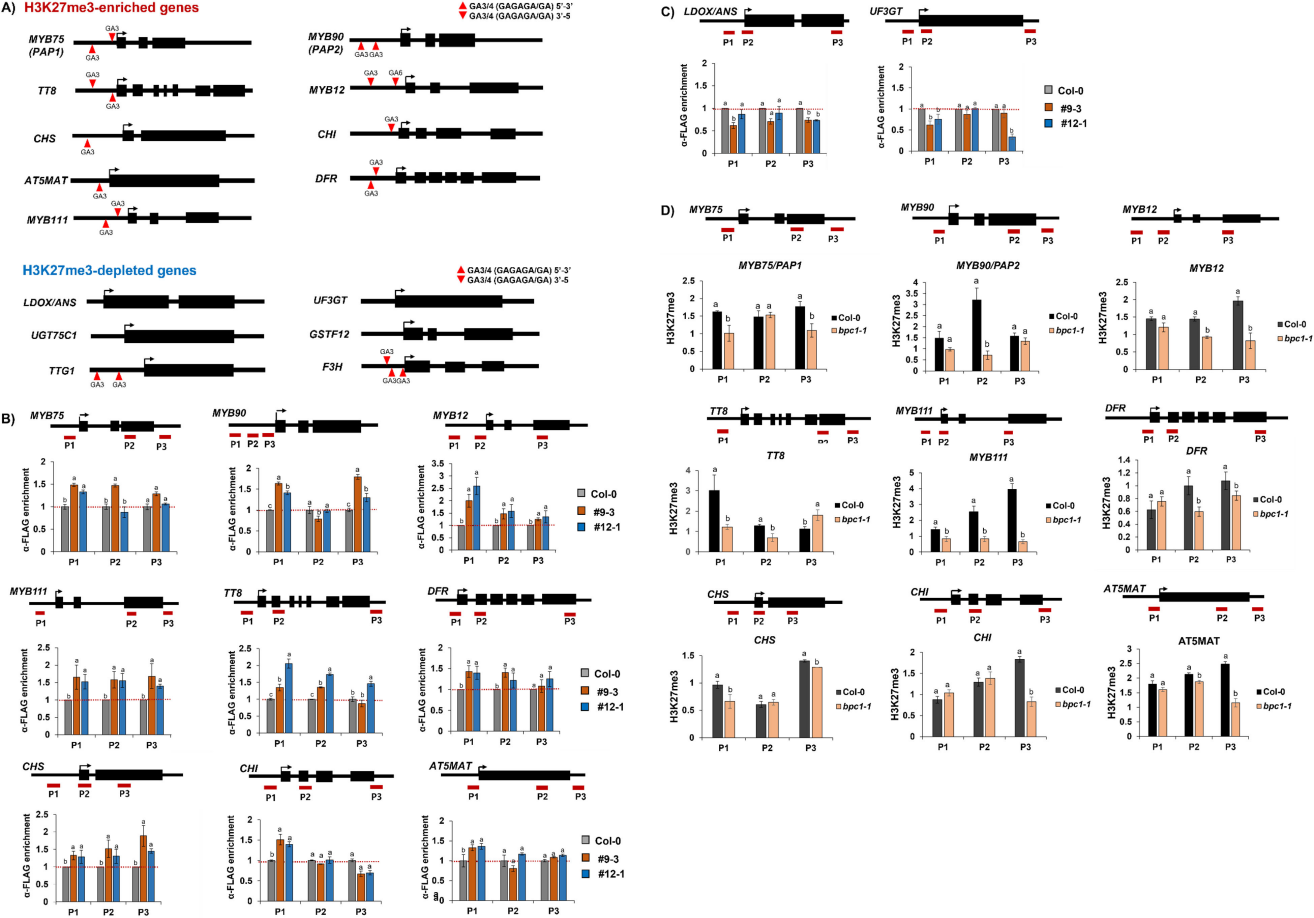
BPC1 binds to the genomic region of H3K27me3-enriched anthocyanin pathway genes. **(A)** Presence or absence of GAGA-type sequence motif(s) in the promoter regions of nine H3K27me3-enriched and six H3K27me3-depleted anthocyanin pathway genes. Black boxes indicate the exon region of the gene. Upward red arrow symbol indicates the sense GAGA-type sequence motif(s). Downward red arrow symbol represents antisense GAGA-type sequence motif(s). **(B)** Result of ChIP-qPCR analysis using α-FLAG antibody (ab205606, Abcam, UK) between the *bpc1–1* mutant and two independent *pBPC1::BPC1-FLAG/bcp1–1* transgenic lines (#9–3 and #12-1) on nine H3K27me3-enriched anthocyanin pathway genes. **(C)** Result of ChIP-qPCR analysis using α-FLAG antibody (ab205606, Abcam, UK) between the *bpc1–1* mutant and two independent *pBPC1::BPC1-FLAG/bcp1–1* transgenic lines on two anthocyanin pathway genes (*LDOX/ANS* and *UF3GT*) among six H3K27me3-depleted anthocyanin pathway genes. None of these genes did not exhibit significant enrichment of BPC1-FLAG. **(D)** Result of ChIP-qPCR analysis using α-H3K27me3 between Col-0 and *bpc1–1* mutant on nine H3K27me3-enriched anthocyanin pathway genes. (**B–D)** One-week-old seedlings were cross-linked at ZT4 time point. Upper panel: A schematic diagram indicating the locations of amplicons (horizontal red lines) used in the ChIP-qPCR assay throughout the genomic region of each anthocyanin pathway gene. The amplicon-amplifying gene body region of reference gene *PP2A* (AT1G69960) was used as a control. Bottom panel: Result of ChIP-qPCR. Precipitated and input DNA from ChIP assay using antibody against GFP were used for qPCR, then the relative enrichment was determined by comparing to the values of *PP2A* (set as 1). Horizontal red dashed line indicates the normalized immunoprecipitated level obtained with the anti-FLAG antibody in the *bpc1–1* mutant. Each result represents the mean ± standard deviation (SD) of three independent biological replicates (n=3). Different letters represent significant differences (*P* < 0.05) determined by one-way analysis of variance (ANOVA) with Tukey’s *post-hoc* test. .

Previous studies have shown that BPC1 recognizes and binds to GAGA-type sequence motifs through coordinating the epigenetic suppression by PRC2 complex (Petrella et al., 2020; Kooiker et al., 2005). To determine whether BPC1 functions as a DNA-binding regulator in the control of anthocyanin biosynthesis, we first examined the presence of GAGA-type motifs in the promoter regions of anthocyanin pathway genes. We found that 11 of 15 genes (73%) contain at least one GAGA-type motif within their promoters (**Figure 3A**). Notably, all nine H3K27me3-enriched genes were included among these 11 genes. To assess direct binding of BPC1 to these loci, we generated a BPC1-3×FLAG transgene driven by its native promoter and introduced it into the *bpc1–1* mutant background. Two independent complemented transgenic lines (#9–3 and #12-1), each harboring a single-copy BPC1-FLAG insertion (referred to as *pBPC1::BPC1-FLAG/bpc1-1*), were isolated (**Supplementary Figure S2**) and used for subsequent ChIP-qPCR analyses. ChIP-qPCR using an anti-FLAG antibody revealed significant enrichment of BPC1-FLAG at the promoter regions of all nine H3K27me3-enriched anthocyanin pathway genes in both transgenic lines, compared with non-transgenic Col-0 (**Figure 3B**). In contrast, no appreciable enrichment of BPC1-FLAG was detected at the promoters of the two tested H3K27me3-depleted genes, *LDOX/ANS* and *UF3GT* (**Figure 3C**), supporting the notion that BPC1 preferentially associates with H3K27me3-enriched loci.

To further investigate the role of BPC1 in establishing the repressive histone mark H3K27me3, we performed H3K27me3 ChIP-qPCR in Col-0 and the *bpc1–1* mutant. Levels of H3K27me3 at all nine H3K27me3-enriched anthocyanin pathway genes were markedly reduced in the *bpc1–1* mutant relative to Col-0 (**Figure 3D**). Together, these results demonstrate that BPC1 directly binds to the promoters of anthocyanin pathway genes and is required for proper H3K27me3 deposition at these loci, thereby contributing to PRC2-mediated epigenetic repression of anthocyanin biosynthesis in *Arabidopsis*.

### BPC1 is not essentially required for the PRC1-mediated suppression of anthocyanin biosynthetic genes

Given that PRC2 closely cooperates with the PRC1 complex to repress target gene expression in *Arabidopsis* (Kim and Sung, 2014), we first performed RT-qPCR analysis in Col-0 and the *bmi1a;bmi1b* double mutant, which lacks functional BMI1a and BMI1b, two core components of the PRC1 complex. A substantial number of anthocyanin pathway genes, six genes (*MYB75/PAP1, MYB90/PAP2, MYB111, MYB12, DFR* and *AT5MAT*) out of nine H3K27me3-enriched genes were significantly de-repressed in the *bmi1a;bmi1b* mutant compared with Col-0 (**Supplementary Figure S3A**). To further validate this observation, we analyzed publicly available RNA-seq data comparing Col-0 and the *bmi1a;bmi1b;bmi1c* (*bmi1abc*) triple mutant, a loss-of-function mutant lacking all three BMI1 paralogs, which are critical components of the PRC1 complex in *Arabidopsis*. Consistent with our RT-qPCR results, a large proportion of H3K27me3-enriched genes including *MYB90, CHS, CHI, MYB12*, and *MYB111* were significantly upregulated in the *bmi1abc* mutant relative to Col-0 (**Supplementary Figure S3B**). In contrast, H3K27me3-depleted genes showed little or no transcriptional activation in the *bmi1abc* mutant, with the exception of *F3H*, which exhibited an increased expression compared with Col-0 (**Supplementary Figure S3C**). Next, we quantified anthocyanin accumulation in *Col-0* and the *bmi1a;bmi1b* (*bmi1ab*) double mutant. The *bmi1ab* mutant exhibited a pronounced increase in anthocyanin content compared with Col-0 (**Supplementary Figures S3D, E**). These results suggest that, in addition to the PRC2 complex, the BMI1-containing PRC1 complex also contributes to the epigenetic repression of anthocyanin pathway genes.

Because the BMI1-containing PRC1 complex represses gene expression by directly catalyzing mono-ubiquitination of histone H2A (H2Aub1), a repressive chromatin mark at target loci (Cao et al., 2005; Zhou et al., 2017; Kim and Sung, 2014), we analyzed publicly available H2Aub1 ChIP-seq datasets comparing Col-0 and the *bmi1a;bmi1b;bmi1c* (*bmi1abc*) triple mutant, a loss-of-function mutant lacking all three BMI1 paralogs in *Arabidopsis* (Baile et al., 2021). Consistent with our RT–qPCR results, all nine H3K27me3-enriched anthocyanin pathway genes showed a significant reduction in H2Aub1 levels in the *bmi1abc* mutant compared with Col-0 (**Supplementary Figure S4A**). These results indicate that the PRC1 complex is also involved in the suppression of anthocyanin pathway genes. Finally, we investigated whether BPC1 is required for PRC1-mediated repression of anthocyanin pathway genes by performing ChIP-qPCR analysis using an anti-H2Aub1 antibody in Col-0 and *bpc1–1* mutant seedlings. However, no significant differences in H2Aub1 enrichment were detected at any of the nine H3K27me3-enriched genes in the *bpc1–1* mutant relative to Col-0 (**Supplementary Figure S4B**). These results suggest that, although BPC1 is required for PRC2-dependent repression of anthocyanin pathway genes, it is not essential for PRC1-mediated regulation of these genes in *Arabidopsis*.

### HY5 directly regulates multiple regulatory and structural genes in the anthocyanin biosynthetic pathway

**Figure 4:**
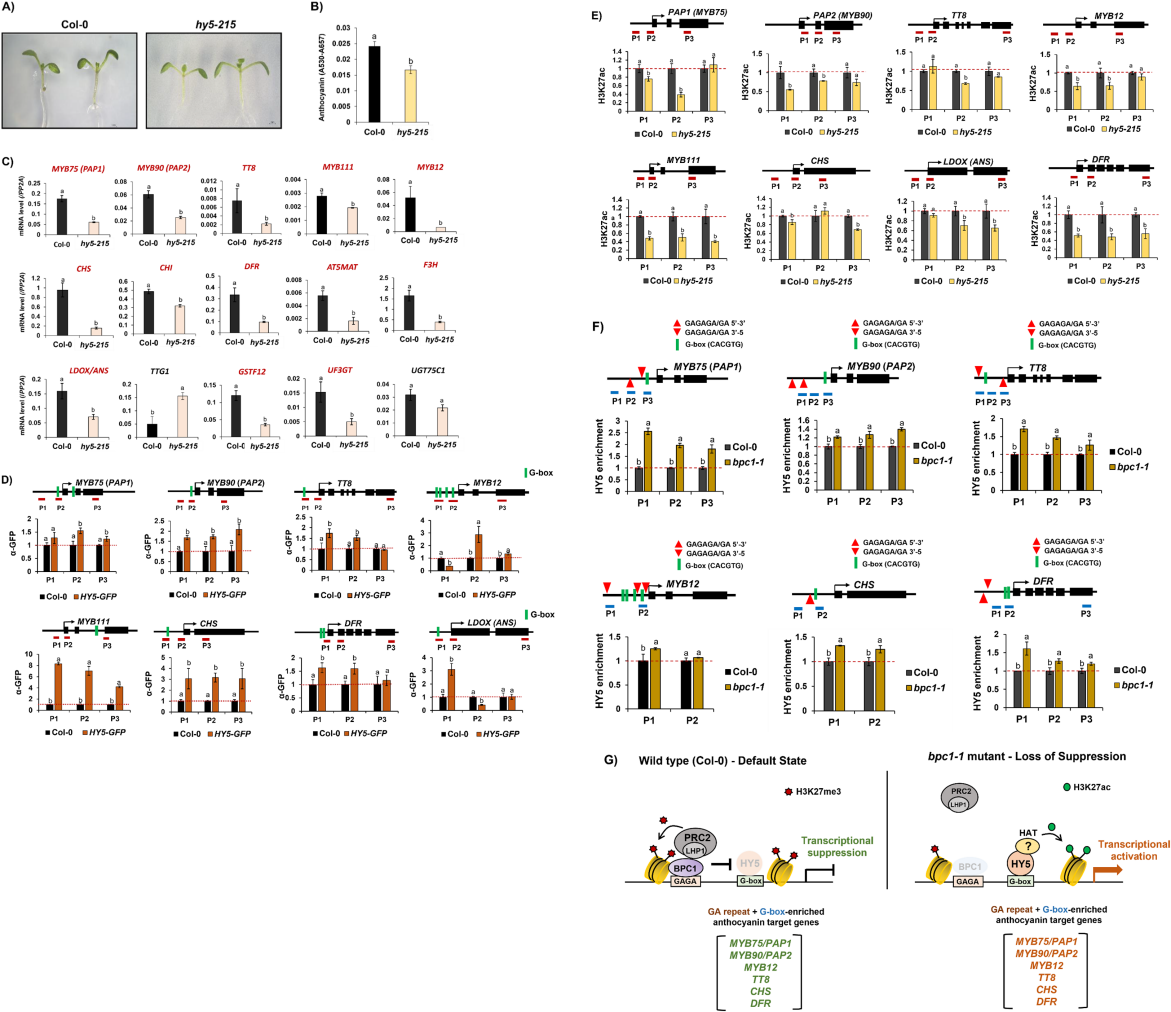
BPC1-mediated H3K27me3 and HY5-mediated H3K27ac oppositely modulate expression of MYB TFs and biosynthetic genes involved in the production of anthocyanin in *Arabidopsis*. **(A)** A representative image showing amounts of anthocyanin between Col-0 and the *hy5–215* mutant grown for one week in the Murashige and Skoog (MS) agar media containing 3% sucrose. Scale bar = 1 mm **(B)** Quantification of anthocyanin between Col-0 and the *hy5–215* mutant grown for one week in the Murashige and Skoog (MS) agar media containing 3% sucrose. Different letters represent significant differences (*P* < 0.05) determined by one-way analysis of variance (ANOVA) with Tukey’s *post-hoc* test. **(C)** Result of qRT-PCR on 12 anthocyanin pathway genes between Col-0 and the *hy5–215* mutant. Total 11 genes (92%) out of 12 tested anthocyanin pathway genes were significantly reduced in the *hy5–215* mutant compared to those of Col-0. One-way ANOVA was applied to calculate the statistical significance, and significant difference was indicated in the figures by different letters (p < 0.05). **(D)** Result of ChIP-qPCR analysis using α-GFP antibody (Cat No.: ab290, Abcam, United Kingdom) between the Col-0 and *p35::HY5-GFP/hy5–215* transgenic line on nine anthocyanin pathway genes. Location of G-box motif **(-**CACGTG-) was indicated by vertical skyblue color line in each genomic region.**(E)** Result of ChIP-qPCR analysis using α-H3K27ac antibody (Cat No.: ab4729, Abcam, United Kingdom) between the Col-0 and *hy5–215* mutant on nine anthocyanin pathway genes. **(F)** Result of ChIP-qPCR analysis using α-HY5 antibody (Cat No.: AS12 1867, Agrisera, Sweden) between the Col-0 and *bpc1–1* mutant on six anthocyanin pathway genes which contains both G-box and GA repeat motifs in its promoter region. **(D–F)** One-week-old seedlings were cross-linked at ZT4 time point. Upper panel: A schematic diagram indicating the locations of amplicons (horizontal red lines) used in the ChIP-qPCR assay throughout the genomic region of each anthocyanin pathway gene. The amplicon-amplifying gene body region of reference gene *PP2A* (AT1G69960) was used as a control (Choi et al., 2024). Bottom panel: Result of ChIP-qPCR. Input and precipitated DNA from ChIP assay using antibody against GFP were used for qPCR, then the relative enrichment was determined by comparing to the values of *PP2A* (set as 1). Each result represents the mean ± standard deviation (SD) of three independent biological replicates (n=3). Different letters represent significant differences (*P* < 0.05) determined by one-way analysis of variance (ANOVA) with Tukey’s *post-hoc* test. **(G)** Mechanistic model illustrating the antagonistic regulation of anthocyanin pathway genes by BPC1-mediated suppression and pre-emptive HY5 occupancy under non-stressed conditions. In wild-type (Col-0) plants, BPC1 recognizes and binds to GA-repeat motifs within the promoter regions of six core anthocyanin target genes (*MYB75/PAP1, MYB90/PAP2, MYB12, TT8, CHS/TT4*, and *DFR/TT3*). BPC1 functions as a molecular tether for the Polycomb Repressive Complex 2 (PRC2), including LHP1, facilitating the deposition of the repressive H3K27me3 histone mark to maintain transcriptional suppression as the default state. In the *bpc1–1* mutant, the loss of BPC1-mediated PRC2 recruitment leads to a reduction in H3K27me3 levels at these loci. This altered chromatin state allows HY5 to pre-emptively occupy proximal G-box motifs even under standard, non-stressed growth conditions, thereby promoting transcriptional activation, likely through H3K27ac deposition by a currently unidentified histone acetyltransferase (HAT, indicated by a question mark) and enhancing anthocyanin biosynthesis. The six core target genes containing overlapping or proximal GAGA and G-box elements are highlighted on the bottom to emphasize the site of direct chromatin-based competition between BPC1 and HY5.

Having established that BPC1 represses anthocyanin pathway genes through PRC2-mediated H3K27me3 deposition, we next investigated whether an opposing transcriptional activator acts on the same target genes. The light signaling factor, HY5 plays a positive role in anthocyanin biosynthesis in *Arabidopsis*. Consistent with this function, *hy5–215* mutant seedlings grown on solid MS medium containing 3% sucrose accumulated substantially lower levels of anthocyanin compared with Col-0 (**Figures 4A, B**). Previous studies have demonstrated that HY5 promotes accumulation of anthocyanin by directly activating the R2R3-MYB transcription factor, *MYB75/PAP1* in *Arabidopsis* (Shin et al., 2013; Kim and Sung, 2017). However, whether HY5 directly regulates additional components of the anthocyanin biosynthetic pathway remains unclear. Addressing this question is essential for understanding how light signaling coordinates transcriptional control across the anthocyanin pathway. Thus, we investigated the extent to which HY5 directly associates with anthocyanin pathway genes and how such regulation may intersect with chromatin-based repression mechanisms. We first examined transcript levels of 15 anthocyanin pathway genes between Col-0 and *hy5–215* mutant. Among the 15 anthocyanin pathway genes examined, 13 genes, excluding *TTG1* and *UGT75C1*, were significantly downregulated in the hy5–215 mutant compared with Col-0 (**Figure 4C**).

Previously, it was reported that HY5 encoding bZIP transcription factor preferentially binds to G-box motifs of the target genomic regions (Chattopadhyay et al., 1998; Lee et al., 2007; Zhang et al., 2011). When we analyzed the genomic region of total 15 anthocyanin pathway genes, 11 out of 15 genes contained at least one G-box motif within its promoter or intronic regions (**Supplementary Figure S5**). We assessed whether HY5 directly associates with anthocyanin pathway genes by performing ChIP-qPCR analysis using the *35S::HY5-GFP/hy5–215* transgenic line. In addition to the previously recognized target gene, MYB75/PAP1, significant enrichments of HY5-GFP were also detected at the promoters of additional seven anthocyanin pathway genes including MYB12, MYB111, MYB90/PAP2, TT8, CHS/TT4, DFR/TT3, and LDOX/ANS/TT18 (**Figure 4D**). Thus, we concluded that HY5 directly regulates multiple transcriptional regulators as well as downstream biosynthetic genes involved in the production of anthocyanin in *Arabidopsis*.

Histone acetylation is closely associated with transcriptionally active chromatin and gene activation (Bannister and Kouzarides 2011; Kouzarides 2007). Previous studies have demonstrated that HY5 coordinates histone acetylation at target gene loci to promote transcriptional activation (Benhamed et al., 2006; Chen et al., 2008). To investigate whether histone acetylation is involved in HY5-mediated activation of anthocyanin pathway genes, we performed ChIP-qPCR analysis using an anti-H3K27ac antibody in Col-0 and *hy5–215* mutant seedlings. Notably, eight HY5-enriched anthocyanin pathway genes exhibited significantly reduced H3K27ac levels in the *hy5–215* mutant compared with Col-0 (**Figure 4E**).

Next, we examined whether loss of functional HY5 affects H3K27me3 enrichment at anthocyanin regulatory and biosynthetic genes. To this end, we analyzed a publicly available H3K27me3 ChIP-seq dataset (GSE233271) comparing Col-0 and the *hy5–215* mutant (Wang et al., 2024). Among the nine genes classified as H3K27me3-enriched, no significant changes in H3K27me3 levels were detected in *hy5–215* mutant relative to Col-0, indicating that loss of HY5 does not markedly alter the H3K27me3 chromatin state of anthocyanin pathway genes (**Supplementary Figure S6**). In contrast, among the six H3K27me3-depleted genes, only the F3H locus exhibited a significant increase in H3K27me3 enrichment in *hy5–215* mutant compared with Col-0. Taken together, these results indicate that HY5 is not a major determinant of H3K27me3 deposition at anthocyanin regulatory or biosynthetic gene loci. Instead, our data support a model in which HY5 directly associates with both transcription factor genes and biosynthetic genes of the anthocyanin pathway and promotes their expression primarily through coordination of histone acetylation, rather than through modulation of H3K27me3.

Furthermore, to directly examine the dynamics of BPC1 and HY5 co-occupancy at shared target loci, we performed an additional ChIP-qPCR experiment comparing HY5 chromatin enrichment between Col-0 and the *bpc1–1* mutant. We focused on six anthocyanin pathway genes including *MYB75/PAP1*, *MYB90/PAP2*, *MYB12*, *TT8*, *CHS/TT4*, and *DFR/TT3*, whose promoter regions contain both a G-box motif (the canonical HY5 binding element) and GA-repeat sequences (the BPC1 binding element), making them prime candidates for competitive or antagonistic co-regulation. ChIP-qPCR analysis using an anti-HY5 antibody revealed that all six target genes exhibited significantly higher HY5 enrichment in the *bpc1–1* mutant compared to Col-0 (**Figure 4F**). To exclude the possibility that increased HY5 chromatin occupancy in the *bpc1–1* mutant simply reflects an elevated abundance of HY5, we examined *HY5* expression levels in our RNA-seq dataset. *HY5* mRNA levels were not significantly different among Col-0, *bpc1-1*, and *bpc1-1;bpc2–1* mutants, indicating that the enhanced HY5 binding at shared target loci reflects altered chromatin competition rather than increased *HY5* transcription (**Supplementary Figure S7**). These results demonstrate that loss of *BPC1* leads to increased HY5 occupancy at shared target loci, suggesting that BPC1 normally restricts HY5 binding, either directly through chromatin compaction via H3K27me3 deposition, or indirectly through competition for overlapping *cis*-regulatory elements. Importantly, this opposing occupancy dynamic was observed under standard basal (non-stressed) growth conditions, providing direct *in vivo* evidence for the antagonistic interplay between BPC1 and HY5 in the regulation of anthocyanin biosynthesis.

Based on these results, we came up with a mechanistic model illustrating the antagonistic regulation of anthocyanin pathway genes by BPC1-mediated suppression and pre-emptive HY5 occupancy under non-stressed conditions (**Figure 4G**). In wild-type (Col-0) plants, BPC1 recognizes and binds to GA-repeat motifs within the promoter regions of six core anthocyanin target genes (*MYB75/PAP1, MYB90/PAP2, MYB12, TT8, CHS/TT4*, and *DFR/TT3*). BPC1 functions as a molecular tether for the Polycomb Repressive Complex 2 (PRC2), including LHP1, facilitating the deposition of the repressive H3K27me3 histone mark to maintain transcriptional suppression as the default state. In the *bpc1–1* mutant, the loss of BPC1-mediated PRC2 recruitment leads to a reduction in H3K27me3 levels at these loci. This altered chromatin state allows HY5 to pre-emptively occupy proximal G-box motifs even under standard, non-stressed growth conditions, thereby promoting transcriptional activation, likely through H3K27ac deposition by a currently unidentified histone acetyltransferase (HAT, indicated by a question mark) and enhancing anthocyanin biosynthesis. The six core target genes containing overlapping or proximal GAGA and G-box elements are highlighted on the bottom to emphasize the site of direct chromatin-based competition between BPC1 and HY5. Together, these results indicate that anthocyanin biosynthesis is governed by a dynamic balance between transcriptional activation and chromatin-based repression, mediated by the opposing activities of HY5-mediated histone acetylation and BPC1-associated PRC2-dependent H3K27me3 deposition.

## Discussion

### BPC1 as a Polycomb-associated repressor of anthocyanin biosynthesis

Anthocyanin biosynthesis is a tightly regulated process that integrates developmental cues and environmental signals (Jaakola, 2013). In this study, we identify BPC1 as a key epigenetic repressor of anthocyanin biosynthesis in *Arabidopsis*. Transcriptome profiling of the *bpc1–1* mutant revealed broad transcriptional activation, consistent with the presence of an EAR repression domain in BPC1 and its previously described role as a transcriptional repressor. Notably, GO analysis suggested that genes involved in secondary metabolisms, particularly anthocyanin biosynthesis, were strongly enriched among upregulated targets. It indicates that BPC1 might play a prominent role in restraining defense-related metabolic pathways under non-inductive conditions. Our data extend earlier work linking BPC proteins to Polycomb-mediated gene silencing. While BPC1 was previously shown to repress developmental regulators such as *SEEDSTICK* (*STK*) via PRC2 recruitment (Petrella et al., 2020; Kooiker et al., 2005), our results demonstrate that this mechanism also applies to metabolic gene networks. High portion of anthocyanin regulatory and biosynthetic genes was enriched for H3K27me3 and de-repressed in both *bpc1–1* and *lhp1–4* mutants. Together, these findings establish BPC1 as a sequence-specific factor that targets PRC2-dependent repression to a defined subset of anthocyanin pathway genes.

### Functional partitioning of anthocyanin pathway genes by chromatin state

Interestingly, our study reveals the classification of 15 anthocyanin pathway genes into nine H3K27me3-enriched and six H3K27me3-depleted groups. Approximately 60% of pathway genes including key transcriptional regulators (*MYB75/PAP1*, *MYB90/PAP2, TT8, MYB12*, *MYB111*) and core biosynthetic enzymes (*CHS, CHI, DFR*, and *AT5MAT*) are under direct Polycomb control (**Figure 2A**). In contrast, other downstream enzymes and accessory genes *(GSTF12, LDOX, TTG1, UF3GT, F3H*, and *UGT75C1*) lack H3K27me3 enrichment (**Figure 2B**) and appear to be regulated indirectly through upstream transcription factors. This chromatin-based partitioning likely reflects a hierarchical regulatory strategy in which Polycomb repression acts primarily at regulatory nodes, thereby exerting broad downstream effects on pathway output. Such an arrangement may provide robustness and energetic efficiency, allowing the plant to maintain metabolic quiescence while retaining the capacity for rapid activation when environmental conditions change. Similar forms of hierarchical Polycomb regulation have been extensively documented in developmental gene networks, where PRC2 preferentially targets key regulatory genes, such as transcription factors controlling meristem identity, floral transition, and organ specification, thereby indirectly constraining large downstream gene programs. Our findings suggest that comparable regulatory principles operate in secondary metabolism, in which Polycomb-mediated repression is concentrated at upstream regulatory nodes of the anthocyanin pathway, allowing coordinated control of multiple biosynthetic genes through a limited number of chromatin-modified loci (Mozgova et al., 2015; Baile et al., 2021; Cai et al., 2023; Hayashi et al., 2023).

### Distinct but coordinated roles of PRC2 and PRC1 in pathway repression

Our results further reveal that both PRC2 and PRC1 complexes contribute to repression of anthocyanin biosynthesis, but through partially independent mechanisms. Loss of BMI1 function led to de-repression of many H3K27me3-enriched genes and increased anthocyanin accumulation, accompanied by reduced H2Aub1 levels at these loci (**Supplementary Figures S3**, **S4A**). This supports a model in which PRC1 reinforces PRC2-mediated silencing of anthocyanin pathway genes. Importantly, however, BPC1 was dispensable for PRC1-mediated H2Aub1 deposition (**Supplementary Figure S4B**), indicating that BPC1 specifically functions upstream of PRC2 but not PRC1. This uncoupling suggests that different sequence-specific factors may independently guide PRC2 and PRC1 activities at metabolic genes, adding an additional layer of regulatory flexibility. Such modular recruitment could allow Polycomb-mediated repression to be fine-tuned in response to developmental or environmental signals.

### HY5 promotes anthocyanin biosynthesis through histone acetylation rather than H3K27me3 removal

In contrast to the repressive role of BPC1, our data demonstrate that HY5 acts as a broad transcriptional activator of the anthocyanin biosynthetic network. Beyond its established role in activating *MYB75/PAP1* (Shin et al., 2013), we show that HY5 directly binds and activates multiple regulatory and biosynthetic genes across the pathway. This positions HY5 as a central coordinator that synchronizes transcriptional activation at multiple levels of the anthocyanin gene hierarchy. Mechanistically, HY5-mediated activation is closely associated with histone acetylation, particularly H3K27ac, at target loci. Loss of HY5 resulted in reduced H3K27ac levels without substantially altering H3K27me3 enrichment at most genes, indicating that HY5 does not antagonize Polycomb-mediated repression by erasing repressive marks. Instead, HY5 appears to promote transcription by establishing or maintaining an active chromatin environment, potentially enabling rapid transcriptional induction even at loci that remain partially marked by H3K27me3.

To further test the antagonistic relationship between BPC1 and HY5, future studies should include epistasis analysis through generation and characterization of a *bpc1-1;hy5–215* double mutant. Physiological and chromatin analyses including anthocyanin quantification, qRT-PCR analysis of target gene expression, and H3K27me3/H3K27ac histone occupancy profiling across Col-0, *bpc1-1*, *hy5-215*, and *bpc1-1;hy5–215* would establish whether the two regulators act additively or epistatically at shared genomic loci, and would provide definitive genetic support for the antagonistic model proposed here.

### An antagonistic chromatin-based regulatory model for secondary metabolism

Our findings support a model in which anthocyanin biosynthesis is governed by an antagonistic interplay between chromatin-based repression and activation. Under non-inductive conditions, BPC1 seems to be constantly expressed (**Supplementary Figure S8A**) and acts to recruit PRC2 to deposit H3K27me3 at key regulatory and biosynthetic genes, maintaining the pathway in a repressed state. Under the same non-inductive conditions, HY5 is present at a basal level and directly associates with overlapping anthocyanin pathway genes, promoting their expression through histone acetylation rather than removal of repressive marks (**Supplementary Figure S6**). This basal HY5 occupancy and H3K27ac deposition, demonstrated here under standard growth conditions, likely establishes a poised chromatin state that is competent for rapid transcriptional induction. Stressed condition like exposure to UV light strongly enhance HY5 accumulation and activity (**Supplementary Figure S8B**) (Rizzini et al., 2011; Shin et al., 2013). How this antagonistic chromatin balance shifts under UV-B stress conditions remains an important future direction. This dual-layered regulatory strategy may allow plants to balance metabolic economy with environmental responsiveness, ensuring that energetically costly secondary metabolites are produced only when beneficial. More broadly, our study highlights how sequence-specific transcription factors can interface with opposing chromatin-modifying machineries to dynamically control plant metabolic pathways, providing a conceptual framework for understanding environmentally responsive epigenetic regulation.

## Materials and methods

### Plant materials and growth conditions

Seeds were sterilized with 1.5% bleach solution and stratified for 3 days at 4 °C, then germinated and grown under long day condition (16-h light/8-h dark) at 22 ˚C. The *bpc1-1* (SALK_072966), bmi1a (SALK_112676), and *bmi1b* (CS903261), *lhp1-4* (also referred to as *tfl2-2*, CS3797) were obtained from the ABRC (Arabidopsis Biological Resource Center). The *bmi1a;bmi1b* double mutant was generated by crossing and further isolated by genotyping of T_2_ progenies. The *hy5–215* mutant was previously reported (Oyama et al., 1997).

### Development of transgenic plants

Genomic DNA of *BPC1* (AT2G01930) was amplified by PCR reaction using Solg™ Pfu-X DNA polymerase (SolGent Co., Republic of Korea). Amplified PCR fragment was cloned into pENTR-D-TOPO vector (Thermo Fisher Scientific, USA). Sequence-verified genomic BPC1 fragment was transferred into PW1357 plant expression vector which contains C-terminal 3xFLAG sequence using LR clonase II enzyme (Thermo Fisher Scientific, USA). Sequence-verified construct was transformed into *Agrobacterium* strain *GV3101*, and then used for transformation into *Arabidopsis* plants using floral dipping (Clough and Bent, 1998). Transgenic plants were selected in a half-strength MS media containing appropriate antibiotics. Homozygous T_3_ lines containing a single copy of transgene were selected and used for molecular analyses including qRT-PCR analysis. Information on the primer sequences used for transgene preparation were shown in the **Supplementary Table S1**.

### RNA-seq library preparation and sequencing

Two-week old seedlings were harvested for the extraction of total RNAs. RNAs were isolated using the RNeasy Plant Mini Kit (QIAGEN, USA) according to the manufacturer’s instructions. The quantity and quality of RNA were checked with a Nano-400 (Allsheng Instrument Co., China). RNA-seq libraries were constructed using the TruSeq Stranded mRNA LT Sample Prep Kit (Illumina, Inc., USA). Sequencing was performed using an Illumina NovaSeq6000 platform using the paired-end sequencing (Macrogen Co., Republic of Korea).

### RNA-seq data analyses

Raw fastq files were quality-checked using the FastQC program (www.bioinformatics.babraham.ac.uk/projects/fastqc/). Low quality of reads was filtered out using Trimmomatic (ver 0.36) prior to the mapping to the reference genome. Trimmed high quality reads were only applied for mapping to *Arabidopsis* TAIR10 genome using STAR aligner with default parameters (Dobin et al., 2013). TAIR10 genome was downloaded from EnsemblPlants. To isolate differentially expressed genes between Col-0 and *bpc1–1* mutant, edgeR program (Robinson et al., 2010) was used in the R package (ver 3.6.1). Venn diagrams analysis was performed using a web-based tool, Venny (ver. 2.1) (https://bioinfogp.cnb.csic.es/tools/venny/index2.0.2.html). Aligned reads were converted to bigwig (bw) files to visualize in the platform of the Integrative Genomics Viewer (IGV) (Thorvaldsdottir et al., 2013).

### Quantification of anthocyanins

Seeds were sterilized and placed on half-strength MS media containing 3% sucrose, then stored at 4 °C for 3 days for stratification treatment. After stratification, seeds plates were incubated under continuous white light condition for 7 days (light intensity: 120 μmol m^-2^ s^-1^) at 22°C. Quantify of anthocyanin was measured using a spectrophotometer, (UV-1800, Shimadzu, Japan).

### Total RNA extraction and quantitative real-time PCR analysis

Total RNAs from two-week old seedlings were extracted using RNeasy Plant mini kit (QIAGEN, USA). Residual contaminated DNA was removed by the treatment of DNaseI (NEB, USA) for 30 min. Total 5 µg of purified total RNAs were used for the synthesis of cDNA using EasyScript reverse transcriptase (TransGen Biotech, China). RT-qPCR analysis was performed using FastFACT™ Real-Time PCR Master Mix (BioFACT, Republic of Korea) in a LineGene 9600 Plus Real-Time PCR system (BioER, China). PCR reaction was conducted under the following condition: initial denaturation at 95 °C for 15 min, followed by 50 cycles at 95 °C for 15 sec, 60 °C for 25 sec, and 72 °C 35 sec. *PP2A* (AT1G13320) was used as a reference gene for normalization (Czechowski et al., 2005). Three biological replicates were analyzed for each qRT-PCR analysis. One-way ANOVA with Tukey’s *post-hoc* test was used for statistical analysis. Information on the primer sequences used in the qRT-PCR analysis were shown in the **Supplementary Table S1**.

### Chromatin immunoprecipitation-qPCR analysis

Whole seedlings of *pBPC1::BPC1-3xFLAG/bpc1–1* transgenic plants or *p35S::HY5-GFP/hy5–215* transgenic plants were grown for two weeks under the long day photoperiod (16h day:8h night). Harvested samples were subsequently cross-linked in 1% formaldehyde solution, then finely ground in liquid nitrogen. ChIP assays were performed as previously described (Kim and Sung, 2017). Antibodies of anti-H3K27me3 (07-449, Millipore, USA), anti-FLAG (ab205606, Abcam, UK), anti-H2Aub1 (8240, Cell Signaling Technology, USA) were purchased and used for ChIP assay. Quantitative PCR (qPCR) analysis was performed using FastFACT™ Real-Time PCR Master Mix (BioFACT, Republic of Korea) according to the manufacturer’s instructions. Enrichment of each amplicon was first calculated based on comparison to Ct value of Input DNA sample. Next, relative enrichment was calculated by comparison to reference gene amplicon. ChIP-qPCR analysis was conducted with three technical replicates from at least two biological replicates. PCR reaction was performed on LineGene 9600 Plus (FQD-96A) Real-Time PCR Detection System (BioER, China). Information on primer sequences used for ChIP-qPCR analysis was shown in **Supplementary Table S1**.

### Public RNA-seq and ChIP-seq dataset analyses

Publicly available RNA-seq and ChIP-seq dataset were downloaded from the European Bioinformatics Institute database (https://www.ebi.ac.uk/). For RNA-seq dataset, raw FASTQ reads were trimmed and quality-filtered before aligning to the *A. thaliana* TAIR10 transcriptome. Alignment to *A. thaliana* TAIR10 genome was performed using Tophat2 (Kim et al., 2013). For ChIP-seq dataset, Bowtie2 was first used for the alignment to *A. thaliana* TAIR10 reference genome (Langmead and Salzberg, 2012). Next, peak callings were conducted using MACS2 command (Zhang et al., 2008). Integrative Genomics Viewer (IGV) was used for visualization of aligned reads (Thorvaldsdottir et al., 2013). Public RNA-seq and ChIP-seq data analyzed in this study were listed in **Supplementary Table S2** and **Supplementary Table S3**, respectively.

### Statistical analysis

The significance of differences between the mean values was assessed with one-way ANOVA with Tukey’s *post hoc* test (p<0.05). Standard deviation (± SD) was provided as an error bar to indicate the variations associated with the particular mean values.

## Data Availability

The datasets presented in this study can be found in online repositories. The names of the repository/repositories and accession number(s) can be found below: https://www.ncbi.nlm.nih.gov/, GSE307463.

## Supplementary material

Supplementary Figure S1 Transcriptome analysis between Col-0 and *bpc1–1* mutant. **(A)** Result of correlation heatmap analysis between Col-0 and *bpc1–1* mutant samples. Three biological replicates of Col-0 and *bpc1–1* mutant samples were used for RNA-seq library construction. Low to high transcriptome profile correlation was indicated with yellow to red color, respectively. **(B)** MA plot of the DEGs between Col-0 and *bpc1–1* mutant. The threshold in the MA plot was adjusted to *P* value < 0.05 and |log2foldchange| >1; red dots represent differentially expressed genes (DEGs) and black dots represent genes with no significant difference.

Supplementary Figure S2 Development of two independent *pBPC1::BPC1-FLAG/bpc1–1* transgenic lines (#9–3 and #12-1). **(A)** Phenotypes of Col-0 and two independent *pBPC1::BPC1-FLAG/bpc1–1* transgenic lines (#9–3 and #12-1) grown on solid MS medium containing 3% sucrose are shown. Seedlings were grown under continuous white light condition for 7 days (light intensity: 120 μmol m^-2^ s^-1^) at 22°C prior to harvest. **(B)** Quantification of anthocyanin contents between Col-0, bpc1–1 mutant, and two *pBPC1::BPC1-FLAG/bpc1–1* transgenic lines (#9–3 and #12-1) which have been grown in solid MS media containing 3% sucrose. Compared to increased amounts of anthocyanin in *bpc1–1* mutant, both selected transgenic lines harboring BPC1-FLAG under the native promoter sequence exhibited even lower amounts of anthocyanin than that of Col-0, indicating that BPC1-FLAG completely rescued anthocyanin accumulation. One-way ANOVA was applied to calculate the statistical significance, and significant difference was indicated in the figures by different letters (p < 0.05).

Supplementary Figure 3 Comparison of normalized transcript reads of 15 anthocyanin pathway genes between Col-0 and *bmi1a;bmi1b;bmi1c* (*bmi1abc*) triple mutants. **(A)** Normalized transcript reads of nine H3K27me3-enriched anthocyanin pathway genes between Col-0 and *bmi1abc* triple mutant. (B) Normalized transcript reads of six H3K27me3-depleted anthocyanin pathway genes between Col-0 and *bmi1abc* triple mutant. **(A, B)** Significantly upregulated genes in the *bmi1abc* triple mutant were indicated with the red box on the gene name. Each result represents the mean ± standard deviation (SD) of two independent biological replicates (n=2). Different letters represent significant differences (*P* < 0.05) determined by one-way analysis of variance (ANOVA) with Tukey’s *post-hoc* test. Detailed information of public RNA-seq dataset between Col-0 and *bmi1abc* triple mutant was shown in the **Supplementary Table S2**. **(C)** Result of qRT-PCR on nine H3K27me3-enriched anthocyanin pathway genes between Col-0 and *bmi1a;bmi1b* (*bmi1a;b*) double mutant. Six genes (60%) out of 10 H3K27me3-enriched anthocyanin pathway genes exhibited upregulated expression in the *bmi1a;b* mutant compared to those of Col-0. **(D)** Result of qRT-PCR on three H3K27me3-depleted anthocyanin pathway genes between Col-0 and the *bmi1a;b* mutant. All of three H3K27me3-depleted anthocyanin pathway genes were also highly upregulated in the *bmi1a;b* mutant compared to those of Col-0. **(C, D)** One-way ANOVA was applied to calculate the statistical significance, and significant difference was indicated in the figures by different letters (p < 0.05). **(E)** Representative image showing amounts of anthocyanin between Col-0 and *lhp1–4* mutant grown for one week in the Murashige and Skoog (MS) agar media containing 3% sucrose. Scale bar = 1cm. **(F)** Quantification of anthocyanin between Col-0 and *bmi1a;bmi1b* (*bmi1a;1b*) double mutant grown for one week in the Murashige and Skoog (MS) agar media containing 3% sucrose.

Supplementary Figure S4 Loss of *BPC1* does not significantly affect H2Aub1 enrichment on H3K27me3-enriched anthocyanin pathway genes. **(A)** Genomic browser view illustrations showing aligned ChIP-seq reads of H2Aub1 on nine H3K27me3-enriched anthocyanin pathway genes between Col-0 and *bmi1a;bmi1b;bmi1c* (*bmi1abc*) triple mutant. Aligned H2Aub1 ChIP-seq reads in Col-0 and the *bmi1abc* mutant background were presented with black and blue color. Enrichment of H2Aub1 of anthocyanin pathway genes were reduced in the *bmi1abc* mutant compared to levels of Col-0 (indicated with dotted red boxes). Read coverage normalized by total number of mapped reads are indicated at the top left or right corner of each track in bracket. Information of public ChIP-seq dataset used in this analysis was shown in the **Supplementary Table S3**. **(B)** Result of ChIP-qPCR analysis using α-H2Aub1 between Col-0 and *bpc1–1* mutant on nine H3K27me3-enriched anthocyanin pathway genes. Each result represents the mean ± standard deviation (SD) of three independent biological replicates (n=3). Different letters represent significant differences (*P* < 0.05) determined by one-way analysis of variance (ANOVA) with Tukey’s *post-hoc* test.

Supplementary Figure S5 Presence or absence of the G-box (CACGTG) motif in the promoter regions of 15 anthocyanin pathway genes. Black boxes represent exonic regions, and vertical green lines indicate the positions of G-box motifs. Eleven of the fifteen anthocyanin pathway genes contain at least one G-box motif within their promoter regions. The start codon of each gene is indicated by a right-directed arrow.

Supplementary Figure S6 Genome browser views showing normalized H3K27me3 ChIP-seq read distributions at nine H3K27me3-enriched genes **(A)** and six H3K27me3-depleted genes **(B)** in Col-0 and the *hy5–215* mutant. **(A)** Aligned H3K27me3 ChIP-seq reads from Col-0 and *hy5–215* are shown in sky blue and moss green, respectively, while corresponding input DNA controls are shown in black. H3K27me3 enrichment at the nine H3K27me3-enriched anthocyanin pathway genes was not significantly altered in *hy5–215* compared with Col-0. **(B)** In case of six H3K27me3-depleted genes, only *F3H* exhibited increased H3K27me3 enrichment in *hy5–215* relative to Col-0 (highlighted by a red box), whereas the remaining genes showed no detectable changes. Details of the public ChIP-seq dataset used in this analysis are provided in **Supplementary Table S3**.

Supplementary Figure S7 Normalized transcript levels of *HY5* (AT5G11260) in wild-type (Col-0), *bpc1-1*, and *bpc1-1;bpc2–1* mutants. Data were obtained from our RNA-seq analysis of two-week-old seedlings grown under standard, non-stressed conditions. Transcript abundance is expressed as normalized reads from three independent biological replicates. Significant difference (*P* < 0.05) was determined by one-way ANOVA analysis with Tukey’s *post-hoc* test.

Supplementary Figure S8 Expression profile of *BPC1* **(A)** and *HY5* **(B)** under different stresses, obtained using the eFP Browser web tool (https://bar.utoronto.ca/eplant/). Expression of *BPC1* remains largely constant even under diverse abiotic stresses. In contrast, *HY5* is significantly and transiently induced by exposure to UV-B stress at 0.5 and 1 hour (highlighted by the red box). The scale bar in the lower left corner indicates expression intensity, with high expression shown in red and low expression in yellow.

Supplementary Table S1 Information on the primers used in this study.

Supplementary Table S2 List of public available RNA-seq dataset analyzed in this study.

Supplementary Table S3 List of public available ChIP-seq dataset analyzed in this study.
