# Supplementary material for: Antagonistic regulation of anthocyanin biosynthesis by HY5 and BPC1 in Arabidopsis thaliana
Source: Front Plant Sci. 2026 May 18;17:1826058. doi: 10.3389/fpls.2026.1826058 (PMC13223105; doi:10.3389/fpls.2026.1826058)
Supplement: Supplementary Figure S1 — Transcriptome analysis between Col-0 and bpc1–1 mutant. (A) Result of correlation heatmap analysis between Col-0 and bpc1–1 mutant samples. Three biological replicates of Col-0 and bpc1–1 mutant samples were used for RNA-seq library construction. Low to high transcriptome profile correlation was indicated with yellow to red color, respectively. (B) MA plot of the DEGs between Col-0 and bpc1–1 mutant. The threshold in the MA plot was adjusted to P value < 0.05 and |log2foldchange| >1; red dots represent differentially expressed genes (DEGs) and black dots represent genes with no significant difference. [file DataSheet1.pdf]

Supplementary information

Supplementary Fig. S1

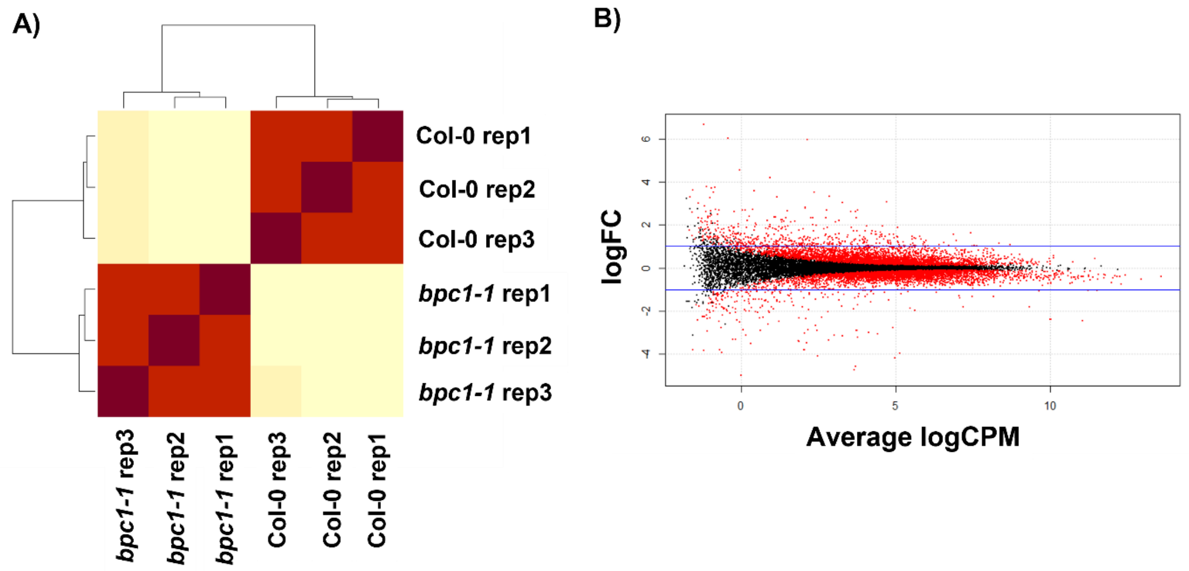

Supplementary Fig. S1. Transcriptome analysis between Col-0 and *bpc1-1* mutant. A) Result of correlation heatmap analysis between Col-0 and *bpc1-1* mutant samples. Three biological replicates of Col-0 and *bpc1-1* mutant samples were used for RNA-seq library construction. Low to high transcriptome profile correlation was indicated with yellow to red color, respectively. **B)** MA plot of the DEGs between Col-0 and *bpc1-1* mutant. The threshold in the MA plot was adjusted to *P* value < 0.05 and |log2foldchange| >1; red dots represent differentially expressed genes (DEGs) and black dots represent genes with no significant difference.

Supplementary Fig. S2.

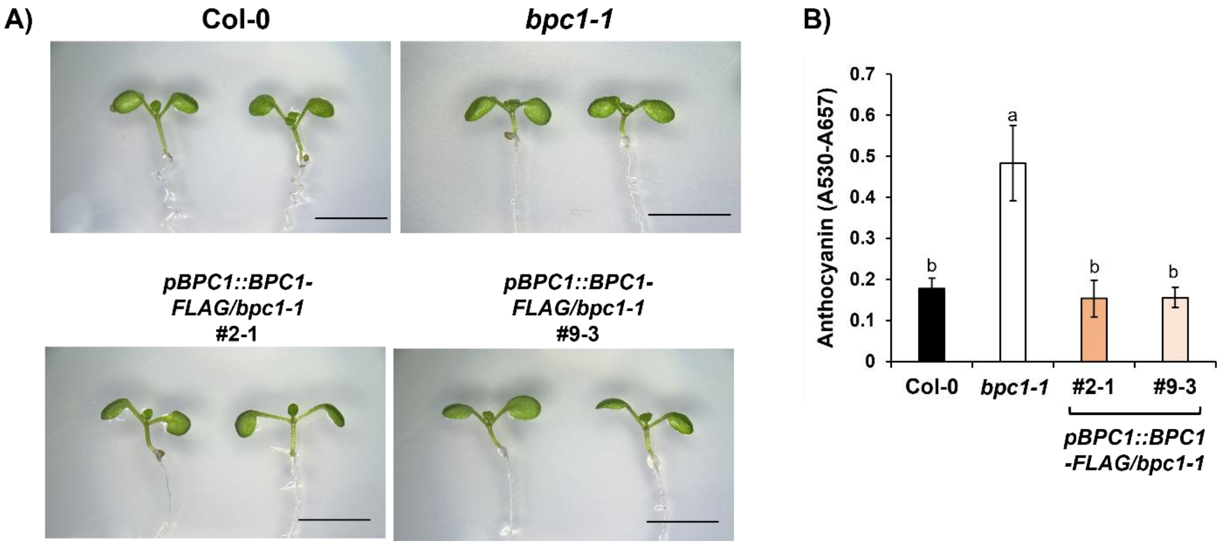

Supplementary Fig. S2. Development of two independent *pBPC1::BPC1-FLAG/bpc1-1* transgenic lines (#9-3 and #12-1). A) Phenotypes of Col-0 and two independent *pBPC1::BPC1-FLAG/bpc1-1* transgenic lines (#9-3 and #12-1) grown on solid MS medium containing 3% sucrose are shown. Seedlings were grown under continuous white light condition for 7 days (light intensity: 120 μmol m^{-2} s^{-1}) at 22℃ prior to harvest. **B)** Quantification of anthocyanin contents between Col-0, bpc1-1 mutant, and two *pBPC1::BPC1-FLAG/bpc1-1* transgenic lines (#9-3 and #12-1) which have been grown in solid MS media containing 3% sucrose. Compared to increased amounts of anthocyanin in *bpc1-1* mutant, both selected transgenic lines harboring BPC1-FLAG under the native promoter sequence exhibited even lower amounts of anthocyanin than that of Col-0, indicating that BPC1-FLAG completely rescued anthocyanin accumulation. One-way ANOVA was applied to calculate the statistical significance, and significant difference was indicated in the figures by different letters ($p < 0.05$).

Supplementary Fig. S3.

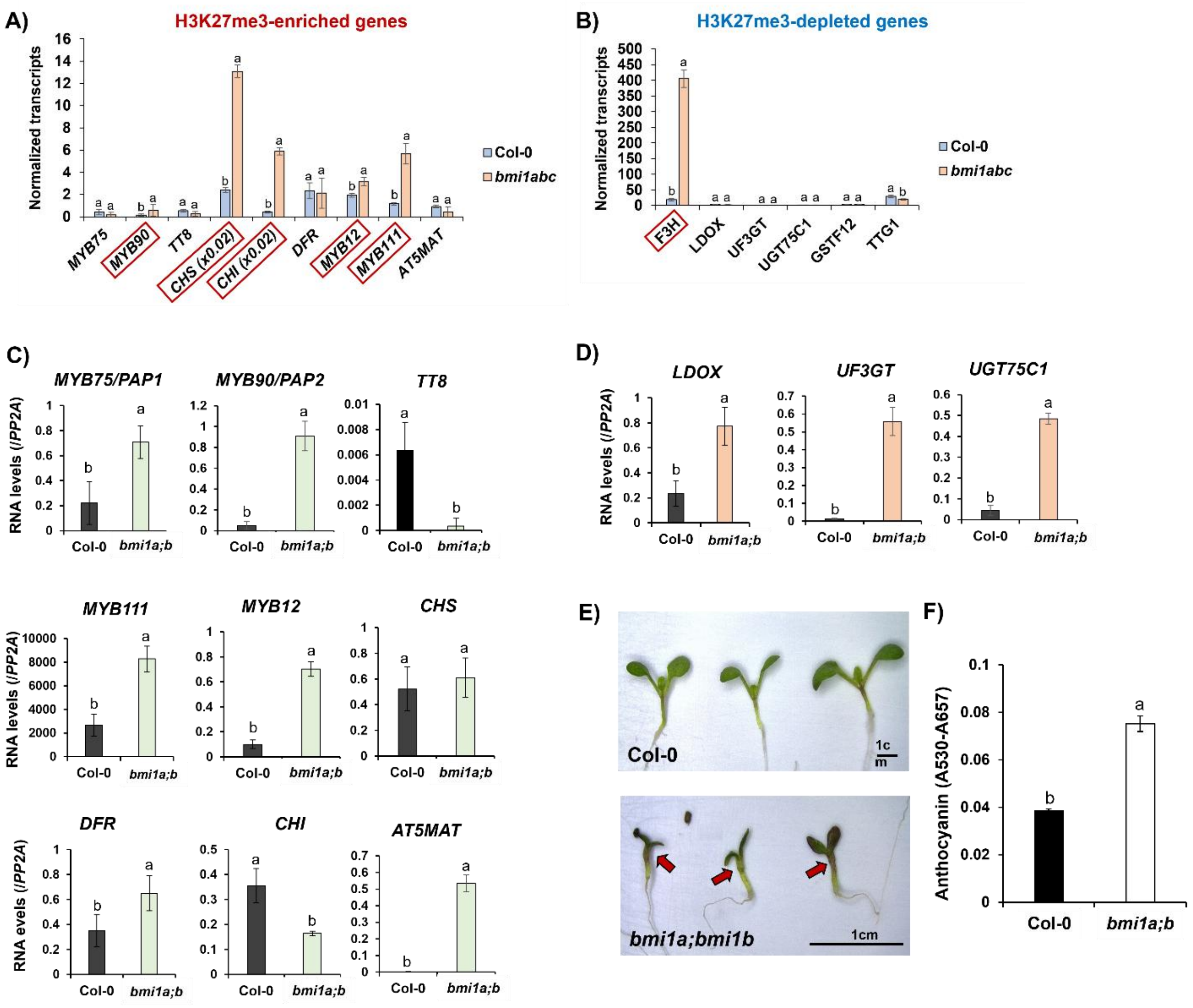

Supplementary Fig. S3. Comparison of normalized transcript reads of 15 anthocyanin pathway genes between Col-0 and *bmi1a;bmi1b;bmi1c* (*bmi1abc*) triple mutants. A) Normalized transcript reads of nine H3K27me3-enriched anthocyanin pathway genes between Col-0 and *bmi1abc* triple mutant. **B)** Normalized transcript reads of six H3K27me3-depleted anthocyanin pathway genes between Col-0 and *bmi1abc* triple mutant. **A**)-**B**) Significantly upregulated genes in the *bmi1abc* triple mutant were indicated with the red box on the gene name. Each result represents the mean ± standard deviation (SD) of two independent biological replicates (n=2). Different letters represent significant differences ($P < 0.05$) determined by one-way analysis of variance (ANOVA) with Tukey's post-hoc test. Detailed information of public RNA-seq dataset between Col-0 and *bmi1abc* triple mutant was shown in the Supplementary Table S2. **C)** Result of qRT-PCR on nine H3K27me3-enriched anthocyanin pathway genes between Col-0 and *bmi1a;bmi1b* (*bmi1a;b*) double mutant. Six genes (60%) out of 10 H3K27me3-enriched

anthocyanin pathway genes exhibited upregulated expression in the *bmi1a;b* mutant compared to those of Col-0. **D)** Result of qRT-PCR on three H3K27me3-depleted anthocyanin pathway genes between Col-0 and the *bmi1a;b* mutant. All of three H3K27me3-depleted anthocyanin pathway genes were also highly upregulated in the *bmi1a;b* mutant compared to those of Col-0. **C)**~**D)** One-way ANOVA was applied to calculate the statistical significance, and significant difference was indicated in the figures by different letters ($p < 0.05$). **E** Representative image showing amounts of anthocyanin between Col-0 and *lhp1-4* mutant grown for one week in the Murashige and Skoog (MS) agar media containing 3% sucrose. Scale bar = 1cm. **F)** Quantification of anthocyanin between Col-0 and *bmi1a;bmi1b* (*bmi1a;1b*) double mutant grown for one week in the Murashige and Skoog (MS) agar media containing 3% sucrose.

Supplementary Fig. S4.

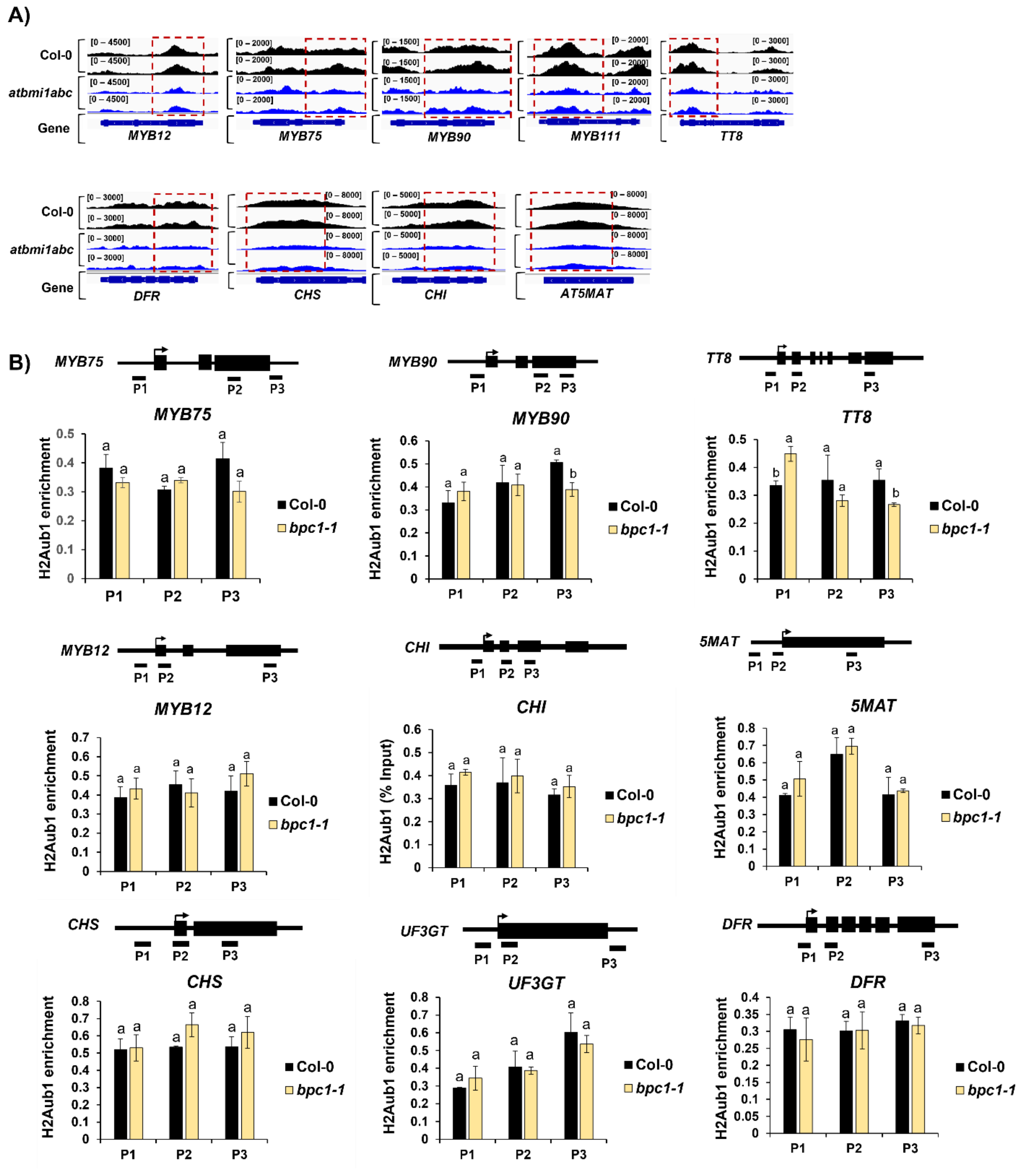

Supplementary Fig. S4. Loss of *BPC1* does not significantly affect H2Aub1 enrichment on H3K27me3-enriched anthocyanin pathway genes. A) Genomic browser view illustrations showing aligned ChIP-seq reads of H2Aub1 on nine H3K27me3-enriched anthocyanin pathway

genes between Col-0 and *bmi1a;bmi1b;bmi1c* (*bmi1abc*) triple mutant. Aligned H2Aub1 ChIP-seq reads in Col-0 and the *bmi1abc* mutant background were presented with black and blue color. Enrichment of H2Aub1 of anthocyanin pathway genes were reduced in the *bmi1abc* mutant compared to levels of Col-0 (indicated with dotted red boxes). Read coverage normalized by total number of mapped reads are indicated at the top left or right corner of each track in bracket. Information of public ChIP-seq dataset used in this analysis was shown in the Supplementary Table S3. **B)** Result of ChIP-qPCR analysis using α-H2Aub1 between Col-0 and *bpc1-1* mutant on nine H3K27me3-enriched anthocyanin pathway genes. Each result represents the mean ± standard deviation (SD) of three independent biological replicates (n=3). Different letters represent significant differences ($P < 0.05$) determined by one-way analysis of variance (ANOVA) with Tukey's post-hoc test.

Supplementary Fig. S5.

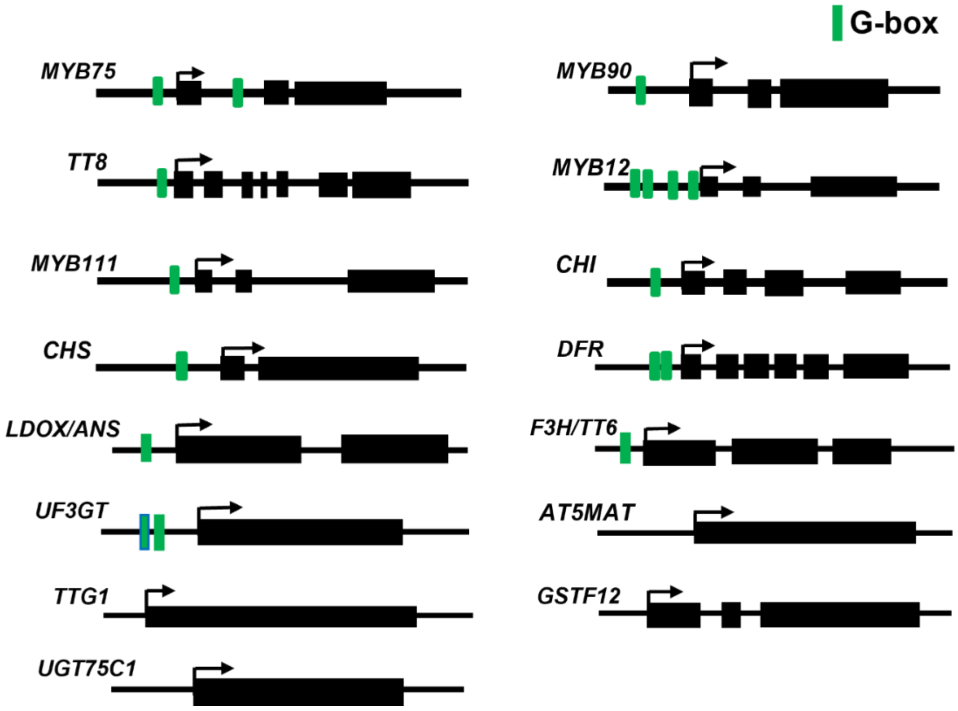

Supplementary Fig. S5. Presence or absence of the G-box (CACGTG) motif in the promoter regions of **15 anthocyanin pathway genes**. **Black boxes** represent exonic regions, and **vertical green lines** indicate the positions of G-box motifs. **Eleven of the fifteen anthocyanin pathway genes** contain at least one G-box motif within their promoter regions. The **start codon** of each gene is indicated by a **right-directed arrow.**

Supplementary Fig. S6.

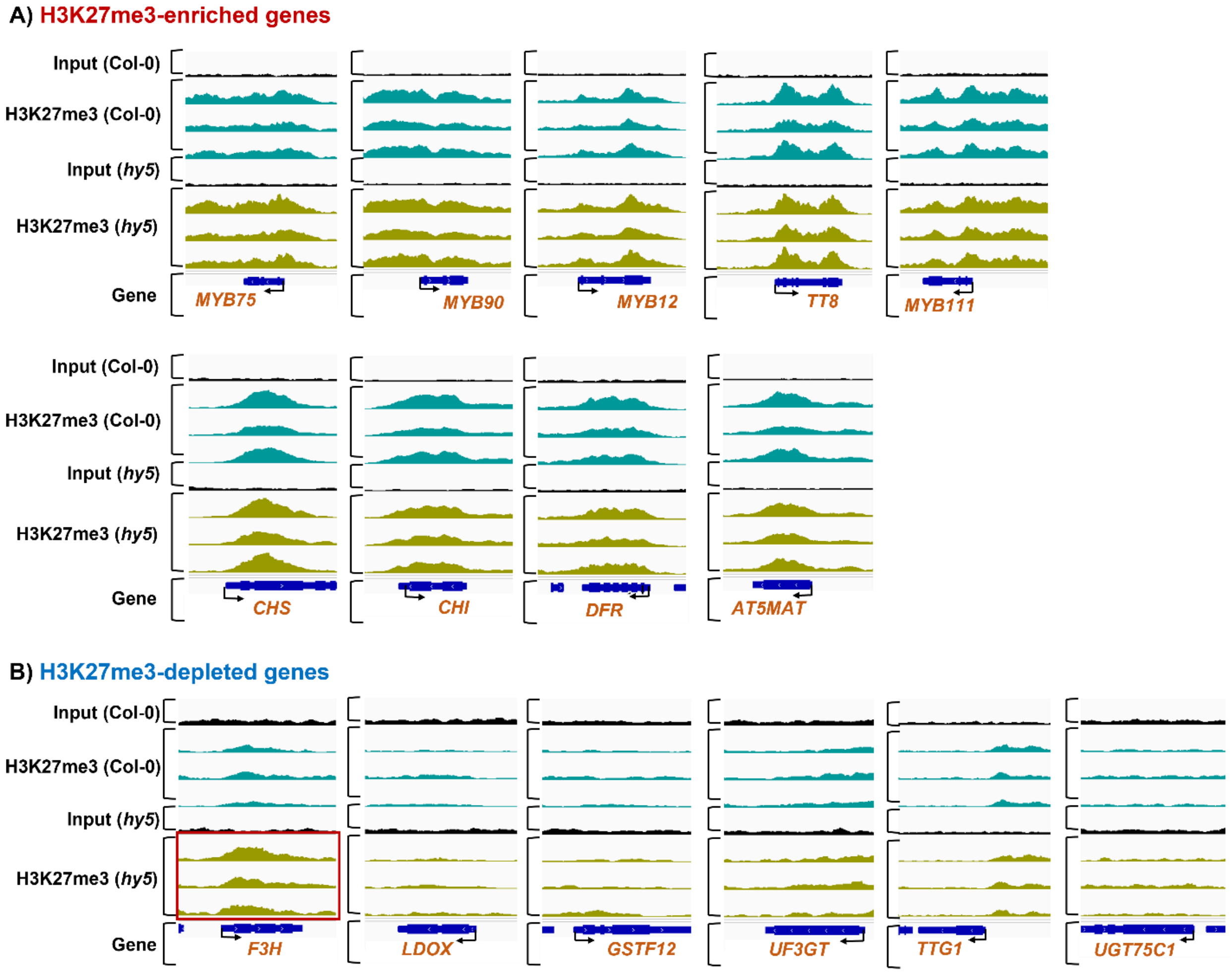

Supplementary Fig. S6. Genome browser views showing **normalized H3K27me3 ChIP-seq read distributions** at **nine H3K27me3-enriched genes** (**A**) and **six H3K27me3-depleted genes** (**B**) in **Col-0** and the ***hy5-215*** mutant. **A**) Aligned H3K27me3 ChIP-seq reads from **Col-0** and ***hy5-215*** are shown in **sky blue** and **moss green**, respectively, while corresponding **input DNA** controls are shown in **black**. H3K27me3 enrichment at the nine H3K27me3-enriched anthocyanin pathway genes was not significantly altered in ***hy5-215*** compared with **Col-0**. **B**) In case of six H3K27me3-depleted genes, only ***F3H*** exhibited increased H3K27me3 enrichment in ***hy5-215*** relative to **Col-0** (highlighted by a **red box**), whereas the remaining genes showed no detectable changes. Details of the public ChIP-seq dataset used in this analysis are provided in **Supplementary Table S3**.

Supplementary Fig. S7.

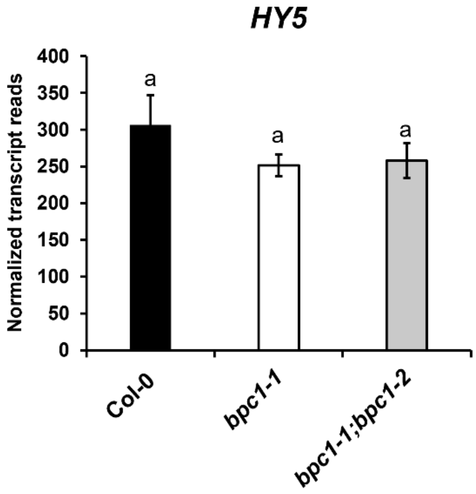

Supplementary Fig. S7. Normalized transcript levels of *HY5* (AT5G11260) in wild-type (Col-0), *bpc1-1*, and *bpc1-1;bpc2-1* mutants. Data were obtained from our RNA-seq analysis of two-week-old seedlings grown under standard, non-stressed conditions. Transcript abundance is expressed as normalized reads from three independent biological replicates. Significant difference ($P < 0.05$) was determined by one-way ANOVA analysis with Tukey's post-hoc test.

Supplementary Fig. S8.

A) Expression profiles of *BPC1* in different stresses

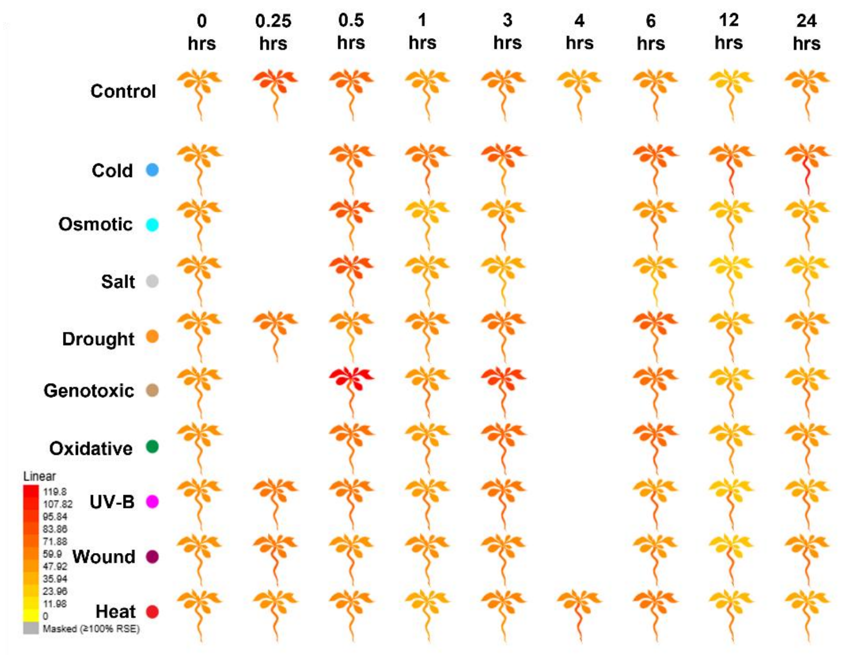

B) Expression profiles of *HY5* in different stresses

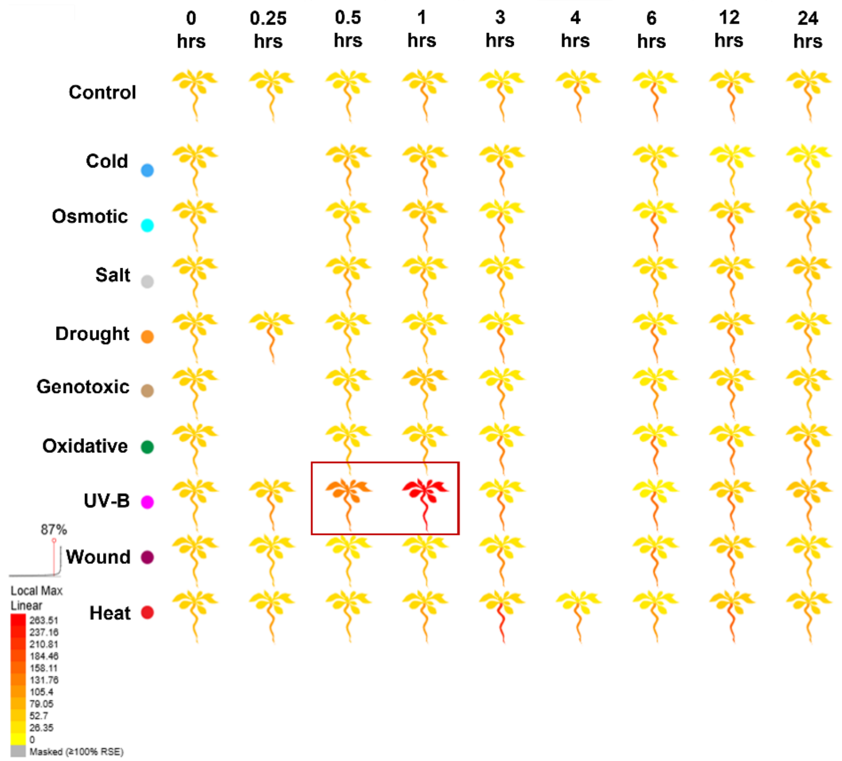

Supplementary Fig. S8. Expression profile of *BPC1* (**A**) and *HY5* (**B**) under different stresses, obtained using the eFP Browser web tool (https://bar.utoronto.ca/eplant/). Expression of *BPC1* remains largely constant even under diverse abiotic stresses. In contrast, *HY5* is significantly and transiently induced by exposure to UV-B stress at 0.5 and 1 hour (highlighted by the red box). The scale bar in the lower left corner indicates expression intensity, with high expression shown in red and low expression in yellow.

Supplementary Table S1. Information on the primers used in this study.

For RT-qPCR analysis

Primer name	Primer sequence (5'-3')
MYB75_F	GTTGGCTCGGGTAATAGTCTCGATG
MYB75_R	GCGGCTTCTCTTTGAACCCAAAC
MYB90_F	TGGCATCAAGTTCCTTTGAGAGCTG
MYB90_R	CATCATTAGCGGTCCGACCAGG
TT8_F	TGATGGTGTTGTGGAACTAGGCACA
TT8_R	TCCCGCGGTAGCCTCTTATCTTTAG
DFR_F	AGCCGCCAAGGGACGTTATATTTG
DFR_R	CCGGGAGAAAACCCTTTTGACGA
LDOX_F	GCCACTGGAAAGATTCAAGGCTATG
LDOX_R	CACTTCGCGTACTCACTCGTTGC
GSTF12_F	GCGAGATACTACGCTACCAAGTTCGCG
GSTF12_R	CCTCGACCAAAACGACGTCACATTTC
5MAT_F	CCGGTTTCCGACGAATCAGC
5MAT_R	ACGGTACTAGATCGTGTGGCAG
UF3GT_F	GTAGTCTTACGTCCGCACGAAGC
UF3GT_R	GCCTTCTGTCTCACGGCAAGT
UGT75C1_F	CTACTCTGTTCTCGTCCCGTGG
UGT75C1_R	GTGGTGATCAGTGGCAGTTTCG
MYB12_F	GGAGCCAGAGGGTTCTCGG
MYB12_R	GCCACGAGACCATTGAGTCGG
MYB111_F	CCTAAGAAAGCTGGATTGTTGAGATGTGG
MYB111_R	CTGTTGCCGAGAAGGGAATGCAAC
CHS_F	CTGCAGGCATCTTGGCTATTGG
CHS_R	TGACGTTTCCGAATTGTCGACTTG
CHI_F	GATAAACTTCTCAAACGCACCGG
CHI_R	TAAATTCGTGATCTTCACCGTCATTGG
F3H_F	GGAGGATTCATCGTCTCTAGTCACC
F3H_R	CAAGCCAAACTCATAAGCCTCTCAC
GL3_F	CGCTCATACGGCGGATAGTAAAGTG
GL3_R	CAGGGGCTTCGAGGAATGATGTC
PP2A_F	TATCGGATGACGATTCTTCGTGCAG
PP2A_R	GCTTGGTCGACTATCGGAATGAGAG

For ChIP-qPCR analysis

Primer name	Primer sequence (5'-3')
MYB75_F1 H3K27me3	GGATGGAGATACTTGCTAGATCCTATG
MYB75_R1 H3K27me3	GGTCTTATAGAGAGTGTGAGTATGTGAG
MYB75_F2 H3K27me3	GAACACTCATCTGAGTAAGAAACATGAAC
MYB75_R2 H3K27me3	CATTGAGATGGTTGCAGTCGTTG
MYB75_F3 H3K27me3	AGTACCAAACCTTCTCTACCGAC
MYB75_R3 H3K27me3	GAGAATCTGCACGTTAACAATCTATGC
MYB90_F1 H3K27me3	GATGGTTGAACTATTTGAAGCCAAGTA
MYB90_R1 H3K27me3	GCAATCGACCAGCAATCAAGG
MYB90_F2 H3K27me3	CAGCCATCTCAATGGTCTGCCAG
MYB90_R2 H3K27me3	CGCTTCAGGAACAATCGCATCAGC
MYB90_F3 H3K27me3	CTGTTTGGTCTAACCGTTAAAATAATAGC
MYB90_R3 H3K27me3	GTCATTTATTTGTTGCAGAACGAATAGTG
DFR_F1 H3K27me3	TTGGCTTACTTTGTCTCTCTGTTTGGAGG
DFR_R1 H3K27me3	TTAGAAGGACAGTGAATCACTTAATCTCATAAAGTA
DFR_F2 H3K27me3	ACGTACCACACATCTCTTTAGTCCTTCGT
DFR_R2 H3K27me3	GTATGTTCGGTACAAATCTTTGTGATGTGAAGAC
DFR_F3 H3K27me3	CATTCATCACAACGTCTATGCCGCC
DFR_R3 H3K27me3	GTCGTCCAAATGCACATACTGTCCTTG
CHS_F1 H3K27me3	GTGAGAGAAAGAAAACGAGGCGTACC
CHS_R1 H3K27me3	GTTTCTTCACCATACAGGAGGTTTCACTT
CHS_F2 H3K27me3	AACCCACCATTCAATCTTGGTAAGTAACG
CHS_R2 H3K27me3	ATTTGGAAATATACCGGAGAGTGAGAGCT
CHS_F3 H3K27me3	GTCTCCGTCCTTCCGTCAAGC
CHS_R3 H3K27me3	GACCGACGAGGGAGTCAAGG
CHI_F1 H3K27me3	TCCTAACTATGTGCTCTGCATATTTAAGGGCTC
CHI_R1 H3K27me3	GGGGCCTTTGGGGTTAAGTAGAAGAG
CHI_F2 H3K27me3	AGTTGGCGTTTGGAAGTTTGGAACGAT
CHI_R2 H3K27me3	TCCCGATGATAGATTCCAGAACCGC
CHI_F3 H3K27me3	GCAGAAAGATTATCTCAGCTAATGATGAAG
CHI_R3 H3K27me3	CCACTTGAGTACCGCATAAGAAATTAG
5MAT_F1 H3K27me3	GAACTTGAACTAGAAATTCGCCTCC
5MAT_R1 H3K27me3	TGGAGGTTATGGGTCCTTACTTAC
5MAT_F2 H3K27me3	GCTCCGTTTGTCGACTTTTGTC
5MAT_R2 H3K27me3	GTTCTGCTTTCACGTCGAAATCTC
5MAT_F3 H3K27me3	ATTCATTACTATTAGCAAGAATGGTGAGC
5MAT_R3 H3K27me3	GTCCCAAATATAGATAAAATAAACGATCAATCATG
MYB12_F1 H3K27me3	AGATGACCTCTCTTTCTTGAACATATACTTG

MYB12_R1 H3K27me3	TACGCCGGATCCTCCTAGTC
MYB12_F2 H3K27me3	CTCCATTGCCTTGACTTTTGTGG
MYB12_R2 H3K27me3	GGAGAGATCTCCAAGAACCTTCAC
MYB12_F3 H3K27me3	CGATTGGGATTGTGTTTGGCAAG
MYB12_R3 H3K27me3	CGTCGTCATGATCTAACGGTTCTC
GL3_F1 H3K27me3	CCTGTGTGTGGTTTCATGGGATATAGG
GL3_R1 H3K27me3	GAAGCAGAGACAGACCAAAAGATACCAT
GL3_F2 H3K27me3	GGAAGACATGAATGTAATACAATGCGTG
GL3_R2 H3K27me3	CGTACTGAACATAGGCGCGTAAATC
GL3_F3 H3K27me3	GATCGGTTCGTTTGGTAATGAGG
GL3_R3 H3K27me3	GACGGTTAAGCAGAGCAAACC
MYB111_F1 H3K27me3	GGCCTCATAATTGTACTCTTAGGCTTCAATCAG
MYB111_R1 H3K27me3	CGTACGAGATTGATCTTGGATATTTTGTCACTTG
MYB111_F2 H3K27me3	CTCACTCTCTCACTCAAGGGTCCAA
MYB111_R2 H3K27me3	GCCAAGAACCTTCACCATTGGTCTG
MYB111_F3 H3K27me3	AGTTACACATAACTAAACCATGCAATTTTTCTGACGT
MYB111_R3 H3K27me3	TCCTGGTAGATGTGTTGCAATAAGTGACCA
TT8_F1 H3K27me3	CACAAAGATGCACACAAGGAGAAGCA
TT8_R1 H3K27me3	GTACACCTGGTTACTTAGACAAAATCAACATTGA
TT8_F2 H3K27me3	ACGTATACATGCATATTGCAAAAATCAGTGGTC
TT8_R2 H3K27me3	CGGAGATACGAAAACGTGGTAGCTCT
TT8_F3 H3K27me3	GGAGGTGGAGGTTTCCATCATAGAG
TT8_R3 H3K27me3	CCTCTTACTTTCGCCCTTATCTCCG
MYB75_F1 H3K9ac/GFP	CAATTAGTTAGCTTTACAGCATATCATTTCGCCTAG
MYB75_R1 H3K9ac/GFP	AGCATTTTTAGTCGTACTTTTTGAGGACTTGCA
MYB75_F2 H3K9ac/GFP	TTCTCTCTAGGACAAATCAGTAACCAAACAATAAC
MYB75_R2 H3K9ac/GFP	GTATGGTTTCACAAATGATTGGAGGTCTTATAGAG
MYB75_F3 H3K9ac/GFP	GATTACCTGGTCGGACCGCAA
MYB75_R3 H3K9ac/GFP	GGGCATTGAGATGGTTGCAGTCG
MYB90_F1 H3K9ac/GFP	CATGTAACAATGATAAGACGAAATATGAGTTTGTGAACA
MYB90_R1 H3K9ac/GFP	CTAGTAATGATTGGTCTCAGAGGGTTCATG
MYB90_F2 H3K9ac/GFP	AAGCAGAACCGCAAATACATGAAATGTGG
MYB90_R2 H3K9ac/GFP	TAAAAAAGGCCGAGGAATAGGGGGT
MYB90_F3 H3K9ac/GFP	CAGCCATCTCAATGGTCTGCCAG
MYB90_R3 H3K9ac/GFP	CGCTTCAGGAACAATCGCATCAGC
DFR_F1 H3K27ac/GFP	TTGGCTTACTTTGTCTCTCTGTTTGGAGG
DFR_R1 H3K27ac/GFP	TTAGAAGGACAGTGAATCACTTAATCTCATAAAGTA
DFR_F2 H3K27ac/GFP	ACGTACCACACATCTCTTTAGTCCTTCGT
DFR_R2 H3K27ac/GFP	GTATGTTCGGTACAAATCTTTGTGATGTGAAGAC
DFR_F3 H3K27ac/GFP	CATTCATCACAACGTCTATGCCGCC

DFR_R3 H3K27ac/GFP	GTCGTCCAAATGCACATACTGTCCTTG
CHS_F1 H3K27ac/GFP	GTGAGAGAAAGAAAACGAGGCGTACC
CHS_R1 H3K27ac/GFP	GTTTCTTCACCATACAGGAGGTTTCACTT
CHS_F2 H3K27ac/GFP	AACCCACCATTCAATCTTGGTAAGTAACG
CHS_R2 H3K27ac/GFP	ATTTGGAAATATACCGGAGAGTGAGAGCT
CHS_F3 H3K27ac/GFP	GTCTCCGTCCTTCCGTCAAGC
CHS_R3 H3K27ac/GFP	GACCGACGAGGGAGTCAAGG
LDOX_F1 H3K27ac/GFP	GAAAATGTGGTTAGTAGAAGAACTAGTAGAGGTG
LDOX_R1 H3K27ac/GFP	CGGGAGAGGATGGTTGGGTTG
LDOX_F2 H3K27ac/GFP	ATGTCTCTTAGTTCGGTAACAAACTCTTCTAACT
LDOX_R2 H3K27ac/GFP	AAGCTAAAACAGAGCAGAAAACAGAGTAAGAAGT
LDOX_F3 H3K27ac/GFP	GAGGGCAAATGGGTCACTGCA
LDOX_R3 H3K27ac/GFP	CACCATCTCCGGCAACGGC
MYB12_F1 H3K27ac/GFP	CTCGTTTGCTTAATTAGGACCATCTGATGAC
MYB12_R1 H3K27ac/GFP	CCTAGTCCGTTGACCAGATGGTC
MYB12_F2 H3K27ac/GFP	GTAACGTCAATGATCTCGGCACACAC
MYB12_R2 H3K27ac/GFP	GGATTAAAAGAGCTTGCGGGTTCGA
MYB12_F3 H3K27ac/GFP	GGTCATCTACCAGGGAGAACAGAC
MYB12_R3 H3K27ac/GFP	CTACTCGTTCTCCCAAGTCTGCG
MYB111_F1 H3K27ac/GFP	GGCCTCATAATTGTACTCTTAGGCTTCAATCAG
MYB111_R1 H3K27ac/GFP	CGTACGAGATTGATCTTGGATATTTTGTCACTTG
MYB111_F2 H3K27ac/GFP	CTCACTCTCTCACTCAAGGGTCCAA
MYB111_R2 H3K27ac/GFP	GCCAAGAACCTTCACCATTGGTCTG
MYB111_F3 H3K27ac/GFP	AGTTACACATAACTAAACCATGCAATTTTTCTGACGT
MYB111_R3 H3K27ac/GFP	TCCTGGTAGATGTGTTGCAATAAGTGACCA
TT8_F1 H3K27ac/GFP	CACAAAGATGCACACAAGGAGAAGCA
TT8_R1 H3K27ac/GFP	GTACACCTGGTTACTTAGACAAAATCAACATTGA
TT8_F2 H3K27ac/GFP	ACGTATACATGCATATTGCAAAAATCAGTGGTC
TT8_R2 H3K27ac/GFP	CGGAGATACGAAAACGTGGTAGCTCT
TT8_F3 H3K27ac/GFP	GGAGGTGGAGGTTTCCATCATAGAG
TT8_R3 H3K27ac/GFP	CCTCTTACTTTCGCCCTTATCTCCG
F3H_F1 H3K27ac/GFP	GACCTCTTCGTTCGTCAGTCATCAC
F3H_R1 H3K27ac/GFP	GTTGAGCTTAGACTCTCCGGCTAG
F3H_F2 H3K27ac/GFP	GAAGCTATGGGTCTTGAGAAAGAGTCTC
F3H_R2 H3K27ac/GFP	CGACTTGGTCTTGTAGCAGCAAGG
F3H_F3 H3K27ac/GFP	ATCCATAGCCACGTTCCAGAACC
F3H_R3 H3K27ac/GFP	GAGGCGAGCAAGCTCCAAATCTC
CHI_F1 H3K27ac/GFP	TAAAGTTCAAACATATCACAGTAGACGTTTAATTTCGA
CHI_R1 H3K27ac/GFP	GGCCCTTTAATCAGTGTGAAAGCATAAATGGT
CHI_F2 H3K27ac/GFP	TCCTAACTATGTGCTCTGCATATTTAAGGGCTC

CHI_R2 H3K27ac/GFP	GGGGCCTTTGGGGTTAAGTAGAAGAG
CHI_F3 H3K27ac/GFP	AGTTGGCGTTTGGAAGTTTGGAACGAT
CHI_R3 H3K27ac/GFP	TCCCGATGATAGATTCCAGAACCGC
CHI_F4 H3K27ac/GFP	GGACAAGTGAAAATCTAGTGAATTAATGTATTCACCTA
CHI_R4 H3K27ac/GFP	GACTTGCGCATCAAAATTCAATTATAAGGACACTA
AT5MAT_F1 H3K27a/GFP	CCTAGCAGGTGGTTGATATTCTTGGGAG
AT5MAT_R1 H3K27a/GFP	CGGGCTAACTTGGACCACCTCA
AT5MAT_F2 H3K27a/GFP	GACTTTCGGTCACGGTTAAACCCTC
AT5MAT_R2 H3K27a/GFP	CCGGACACAATCCGTTGACCC
AT5MAT_F3 H3K27a/GFP	TGTTTTTAGTCCAATTTAGGTATTAGGTTCTATTGTT
AT5MAT_R3 H3K27a/GFP	CTCGTCATTGAAATGATTTTTCAGAAAGTTGA
MYB75_F1 FLAG	GGATGGAGATACTTGCTAGATCCTATG
MYB75_R1 FLAG	GGTCTTATAGAGAGTGTGAGTATGTGAG
MYB75_F2 FLAG	GAACACTCATCTGAGTAAGAAACATGAAC
MYB75_R2 FLAG	CATTGAGATGGTTGCAGTCGTTG
MYB75_F3 FLAG	AGTACCAAACCTTCTCTACCGAC
MYB75_R3 FLAG	GAGAATCTGCACGTTAACAATCTATGC
MYB90_F1 FLAG	CAAGGATTTGTGTAAGCCATAGTATGTATACC
MYB90_R1 FLAG	CCTCAGAGACTATCTTCTTCACCATCCCATG
MYB90_F2 FLAG	CAGCCATCTCAATGGTCTGCCAG
MYB90_R2 FLAG	CGCTTCAGGAACAATCGCATCAGC
MYB90_F3 FLAG	CTGTTTGGTCTAACCGTTAAAATAATAGC
MYB90_R3 FLAG	GTCATTTATTTGTTGCAGAACGAATAGTG
DFR_F1 FLAG	TTGGCTTACTTTGTCTCTCTGTTTGGAGG
DFR_R1 FLAG	TTAGAAGGACAGTGAATCACTTAATCTCATAAAGTA
DFR_F2 FLAG	GTACAACATCTTCTTGATTTGCCAAAC
DFR_R2 FLAG	CTCAGGATCTTTTGATTCAAAATCCATG
DFR_F3 FLAG	CATTCATCACAACGTCTATGCCGCC
DFR_R3 FLAG	GTCGTCCAAATGCACATACTGTCCTTG
CHS_F1 FLAG	GTGAGAGAAAGAAAACGAGGCGTACC
CHS_R1 FLAG	GTTTCTTCACCATACAGGAGGTTTCACTT
CHS_F2 FLAG	AACCCACCATTCAATCTTGGTAAGTAACG
CHS_R2 FLAG	ATTTGGAAATATACCGGAGAGTGAGAGCT
CHS_F3 FLAG	GTCTCCGTCCTTCCGTCAAGC
CHS_R3 FLAG	GACCGACGAGGGAGTCAAGG
CHI_F1 FLAG	TCCTAACTATGTGCTCTGCATATTTAAGGGCTC
CHI_R1 FLAG	GGGGCCTTTGGGGTTAAGTAGAAGAG
CHI_F2 FLAG	AGTTGGCGTTTGGAAGTTTGGAACGAT
CHI_R2 FLAG	TCCCGATGATAGATTCCAGAACCGC
CHI_F3 FLAG	GCAGAAAGATTATCTCAGCTAATGATGAAG

CHI_R3 FLAG	CCACTTGAGTACCGCATAAGAAATTAG
5MAT_F1 FLAG	CCTAGCAGGTGGTTGATATTCTTGGGAG
5MAT_R1 FLAG	CGGGCTAACTTGGACCACCTCA
5MAT_F2 FLAG	GCTCCGTTTGTCGACTTTTGTC
5MAT_R2 FLAG	GTTCTGCTTTCACGTCGAAATCTC
5MAT_F3 FLAG	ATTCATTACTATTAGCAAGAATGGTGAGC
5MAT_R3 FLAG	GTCCCAAATATAGATAAAATAAACGATCAATCATG
MYB12_F1 FLAG	AGATGACCTCTCTTTCTTGAACATATACTTG
MYB12_R1 FLAG	TACGCCGGATCCTCCTAGTC
MYB12_F2 FLAG	CTCCATTGCCTTGACTTTTGTGG
MYB12_R2 FLAG	GGAGAGATCTCCAAGAACCTTCAC
MYB12_F3 FLAG	CGATTGGGATTGTGTTTGGCAAG
MYB12_R3 FLAG	CGTCGTCATGATCTAACGGTTCTC
MYB111_F1 FLAG	GGCCTCATAATTGTACTCTTAGGCTTCAATCAG
MYB111_R1 FLAG	CGTACGAGATTGATCTTGGATATTTTGTCACTTG
MYB111_F2 FLAG	CTCACTCTCTCACTCAAGGGTCCAA
MYB111_R2 FLAG	GCCAAGAACCTTCACCATTGGTCTG
MYB111_F3 FLAG	AGTTACACATAACTAAACCATGCAATTTTTCTGACGT
MYB111_R3 FLAG	TCCTGGTAGATGTGTTGCAATAAGTGACCA
LDOX_F1 FLAG	CTCTACAATGTCTCTTAGTTCGGTAAC
LDOX_R1 FLAG	CTTAAAAACAGGGGAAAAAACAGAGTAAT
LDOX_F2 FLAG	GTATGCAAACGATCAAGCCACTG
LDOX_R2 FLAG	CACTTGGTGTCTTAGGCCAAATTG
LDOX_F3 FLAG	CATCGTGGGTTGGTGAATAAGG
LDOX_R3 FLAG	GTTCCTTCCCAAACAACTTATGCTC
TT8_F1 FLAG	ACGTATACATGCATATTGCAAAAATCAGTGGTC
TT8_R1 FLAG	CGGAGATACGAAAACGTGGTAGCTCT
TT8_F2 FLAG	GCAATAAAGACGAGGAAGACAACTC
TT8_R2 FLAG	GCACATTAGATAAAACCATTCTGTCTCC
TT8_F3 FLAG	AGCCTCACATGTATTAAGACATCAG
TT8_R3 FLAG	ATACAAAAGTACTAAATTGGACAACGAAC
UF3GT_F1 FLAG	GTTGACTGTGGGATTATTAAGCGTAACG
UF3GT_R1 FLAG	GTAACCTCCTGGTAAGTGCAAACAC
UF3GT_F2 FLAG	GTGCTAAAACCGTTTGCTTCAACATC
UF3GT_R2 FLAG	CTTCGTGCGGACGTAAGACTAC
UF3GT_F3 FLAG	CTTCGGGTGTATCAACCGTCG
UF3GT_R3 FLAG	CGATCTGACAATCACTCATCAACGAC
PP2A_F	GATTATCTTCCACTTACAGCTCTTATTGAGAGTCAG
PP2A_R	TAAAGATCGGATGTTGTCAAGTGTATCTAAAGAAGG
For anti-HY5 ChIP-qPCR	primer sequence (5’-3’)

CHS-ChIP-pro-LP1	GGT CAA CCG GAT TAA AAG AAA ACG G
CHS-ChIP-pro-RP1	CCA TAG GCC CAC ATG GGT TAT G
CHS-ChIP-pro-LP2	CGA GGC GTA CCT AAT GTA ACA CTA C
CHS-ChIP-pro-RP2	CAC CAT ACA GGA GGT TTC ACT TCC
CHS-ChIP-pro-LP3	CAT CTG CCC GTC CAT CTA ACC
CHS-ChIP-pro-RP3	CCA ACT TGG GTT TAT TAG AGT TTG ATG
MYB12-ChIP-pro-LP1	GGA CAA CCA ACC AAA CAA CAA TTA TG
MYB12-ChIP-pro-RP1	CGT GAT GTC TTC TGT GGT CTG ATA G
MYB12-ChIP-pro-LP2	GTG GTT ATC ACT AAC ACG CTG ACG
MYB12-ChIP-pro-RP2	ATC AGA TAG TAA TCG CAC ATT TTT TGT TC
MYB12-ChIP-pro-LP3	CGA ACC CGC AAG CTC TTT TAA TC
MYB12-ChIP-pro-RP3	GTG AGA AAG AGA AGT TAC TGA GTG ACA G
MYB75-ChIP-pro-LP1	CTA CAC CAG GTT TGG GTT CGA GTC
MYB75-ChIP-pro-RP1	GTG TGA GCC CTC TAC TCT TGG TTT C
MYB75-ChIP-pro-LP2	CAC CAA CAA TGA TTC TAA TCG GCA C
MYB75-ChIP-pro-RP2	GGG AGT TAA GAT AGT GTA TAG TAT GTT CAG
MYB75-ChIP-pro-LP3	GGA GAT ACT TGC TAG ATC CTA TGA ACC
MYB75-ChIP-pro-RP3	GAG GTC TTA TAG AGA GTG TGA GTA TGT G
MYB90-ChIP-pro-LP1	CCA TTG TTG TTA GGG TTT TTG GCA AG
MYB90-ChIP-pro-RP1	GCA GGT TAA ATT AGA TTT CGT GGA GTT G
MYB90-ChIP-pro-LP2	GTC CAC GCA TAG CAT TGA TTA GAA CC
MYB90-ChIP-pro-RP2	CGA CCT CTT TTG GTC TCC ACT AG
MYB90-ChIP-pro-LP3	GGA AAA GGT GCT ATG AAC TTT TTC TCT
MYB90-ChIP-pro-RP3	CTT CCC AAA TCC TGG GAG GAC TTA TTG
DFR-ChIP-pro-LP1	AGG TGT CTG ATT TTT AGA TTT CAA TTA AA
DFR-ChIP-pro-RP1	GTG AGA CGT GTG GGT GAC TTC
DFR-ChIP-pro-LP2	ACG TAC CAC ACA TCT CTT TAG TCC TTC GT
DFR-ChIP-pro-RP2	GTA TGT TCG GTA CAA ATC TTT GTG ATG TGA AGA C
DFR-ChIP-pro-LP3	CAT TCA TCA CAA CGT CTA TGC CGC C
DFR-ChIP-pro-RP3	GTC GTC CAA ATG CAC ATA CTG TCC TTG
TT8_pro_LP_1	CAA AGA TGC ACA CAA GGA GAA GC
TT8_pro_RP_1	GAC AAA ATA AAT GTT GCT TGG CTT TTG
TT8_pro_LP_2	GTC AGG TCA ACA ATT CTA CCA TTC C
TT8_pro_RP_2	GAG CAA AGA AAT TGC GTT TTT CAC AT
TT8_pro_LP_3	ATC AGT GGT CCC ATA CCA TTT TAA G
TT8_pro_RP_3	CGT TCC CGG AGA TAC GAA AAC

Supplementary Table S2. List of public available RNA-seq dataset analyzed in this study.

Type	Samples	Accession number
RNA-seq	Col-0 rep1	GSM2367133
	Col-0 rep2	GSM2367134
	bmi1abc rep1	GSM2367135
	bmi1abc rep2	GSM2367136

Supplementary Table S3. List of public available ChIP-seq dataset analyzed in this study.

Type	Samples	Accession number
ChIP-seq	Input	SRR970148
	H3K27me3	SRR1931604
	H3K27me3	SRR1774027
	Input	SRR3087683
	LHP1_GFP	SRR3087684
	H2AK121ub Col-0 rep1	GSM2367137
	H2AK121ub Col-0 rep2	GSM2367138
	H2AK121ub *bmi1abc* rep1	GSM2367139
	H2AK121ub *bmi1abc* rep2	GSM2367140
	Col-0 Input	GSE233271
	H3K27me3 Col-0 rep1	
	H3K27me3 Col-0 rep2	
	H3K27me3 Col-0 rep3	
	hy5-215 Input	
	H3K27me3 *hy5-215* rep1	
	H3K27me3 *hy5-215* rep2	
	H3K27me3 *hy5-215* rep3	
